# Polyphenolics, glucosinolates and isothiocyanates profiling of aerial parts of *Nasturtium officinale* (Watercress)

**DOI:** 10.3389/fpls.2022.998755

**Published:** 2022-11-15

**Authors:** Sotiris Kyriakou, Kyriaki Michailidou, Tom Amery, Kyle Stewart, Paul G. Winyard, Dimitrios T. Trafalis, Rodrigo Franco, Aglaia Pappa, Mihalis I. Panayiotidis

**Affiliations:** ^1^ Department of Cancer Genetics, Therapeutics & Ultrastructural Pathology, The Cyprus Institute of Neurology & Genetics, Nicosia, Cyprus; ^2^ Biostatistics Unit, The Cyprus Institute of Neurology & Genetics, Nicosia, Cyprus; ^3^ The Watercress Company, Dorchester, United Kingdom; ^4^ Watercress Research Limited, Devon, United Kingdom; ^5^ Laboratory of Pharmacology, Medical School, National & Kapodistrian University of Athens, Athens, Greece; ^6^ Redox Biology Centre, University of Nebraska-Lincoln, Lincoln, NE, United States; ^7^ Department of Veterinary Medicine & Biomedical Sciences, University of Nebraska-Lincoln, Lincoln, NE, United States; ^8^ Department of Molecular Biology & Genetics, Democritus University of Thrace, Alexandroupolis, Greece

**Keywords:** watercress, polyphenolic acids, flavonoids, glucosinolates, isothiocyanates, thiourea derivatives, UPLC-ESI-MS/MS

## Abstract

Watercress (*Nasturtium officinale*) is a rich source of secondary metabolites with disease-preventing and/or health-promoting properties. Herein, we have utilized extraction procedures to isolate fractions of polyphenols, glucosinolates and isothiocyanates to determine their identification, and quantification. In doing so, we have utilized reproducible analytical methodologies based on liquid chromatography with tandem mass spectrometry by either positive or negative ion mode. Due to the instability and volatility of isothiocyanates, we followed an ammonia derivatization protocol which converts them into respective ionizable thiourea derivatives. The analytes’ content distribution map was created on watercress flowers, leaves and stems. We have demonstrated that watercress contains significantly higher levels of gluconasturtiin, phenethyl isothiocyanate, quercetin-3-*O*-rutinoside and isorhamnetin, among others, with their content decreasing from flowers (82.11 ± 0.63, 273.89 ± 0.88, 1459.30 ± 12.95 and 289.40 ± 1.37 ng/g of dry extract respectively) to leaves (32.25 ± 0.74, 125.02 ± 0.52, 1197.86 ± 4.24 and 196.47 ± 3.65 ng/g of det extract respectively) to stems (9.20 ± 0.11, 64.7 ± 0.9, 41.02 ± 0.18, 65.67 ± 0.84 ng/g of dry extract respectivbely). Pearson’s correlation analysis has shown that the content of isothiocyanates doesn’t depend only on the bioconversion of individual glucosinolates but also on other glucosinolates of the same group. Overall, we have provided comprehensive analytical data of the major watercress metabolites thereby providing an opportunity to exploit different parts of watercress for potential therapeutic applications.

## 1 Introduction

Watercress (*Nasturtium officinal*e) is a widely and extensively studied perennial cruciferous plant. As a member of the *Brassicaceae* family, it is a rich source of various phytochemicals including polyphenols (phenolic acids, flavonoids and proanthocyanins), pigments (chlorophylls, lycopene and carotenoids), and isothiocyanates (ITCs). In addition to this, watercress is a source of soluble sugars, proteins and vitamins (Zeb, 2015; Ma et al., 2021; Kyriakou et al., 2022a; Kyriakou et al., 2022b).

Glucosinolates (GLs), the precursors of ITCs, belong to of nitrogenous-sulfur- enriched phytochemicals. Most of the time, GLs are overexpressed in cruciferous vegetables and are structurally characterized by the presence of a *β*-D-thioglucosidic bond which is linked to ***D***-glucose (Barba et al., 2016; Prieto et al., 2019). GLs can be segregated into various classes (over 200) according to the different types of groups which are linked to them with the major ones being sulfoxides, methyl sulfites, methyl sulphates, saturated aliphatics, allylics, aromatics and indolyls (Ishida et al., 2014). The bioconversion of GLs into active ITCs is regulated by the presence of an endogenous myrosinase which is activated upon tissue disruption as part of plant defense mechanism (Kyriakou et al., 2022a). However, variations on pH, ferrous ions, ascorbate availability and epithiospecifier protein(s) expression can drive rearrangement into nitriles, thiocyanates, epithionitriles and oxazolidine-2-thiones rather than ITCs (Wentzell and Kliebenstein, 2008; Hanschen and Schreiner, 2017; Wang et al., 2019).

The biochemical importance of watercress supplementation (or its extracts) has been reported numerous times in the literature. For instance, it has been previously suggested that watercress juice controls hyperglycemia by restricting the activity of α-glucosidase while enhances that of lipase and α-amylase (Spínola et al., 2017). In another study, it has been demonstrated that watercress consumption was associated with augmentation of superoxide dismutase and catalase activities with a concomitant reduction of hepatic glutathione (GSH), glutathione reductase (GR), glutathione peroxidase (GPx) and malondialdehyde (MDA) contents in hypercholesterolaemic rats (Azarmehr et al., 2019). In addition, others have demonstrated that the administration of raw watercress increases the accumulation of plasma antioxidants including lutein and β-carotene (Gill et al., 2007). Finally, other studies have shown that watercress extracts enriched in either phenethyl isothiocyanate; PEITC or polyphenolic compounds induce cytotoxicity in various human cancer cell lines including melanoma, prostate, leukemia, cervical, liver, colon, lung, myeloma and breast (Gao et al., 2011; Kim et al., 2011; Sun et al., 2019; Mitsiogianni et al., 2021a; Mitsiogianni et al., 2021b).

To conclude, this study focuses into the descriptive characterization and evaluation of the main phytochemicals (glucosinolates, polyphenolics and isothiocyanates) present in the aerial parts of watercress including watercress flowers, leaves and stems. The information obtained also highlights the possibility of exploitation of watercress by products, such as stems.

## 2 Materials and methods

### 2.1 Reagents

Solvents: Methanol LC-MS, grade ≥ purity 99.9% (34860), water HPLC grade (34877), acetonitrile HPLC grade, purity ≥ 99.9 (34851), were purchased from Honeywell (Medisell Nicosia, Cyprus). Formic acid LC-MS grade (85178) and trifluoroacetic acid LC-MS grade (85183) were purchased from Thermofisher Scientific (G. Georgiou, Nicosia, Cyprus). 2M ammonia solution in isopropanol (392693) was purchased from Sigma Aldrich (Vouros, Nicosia, Cyprus). The analytical standards: gluociberin potassium salt (2513S), glucoraphanin potassium salt (2509S), glucocamelinin potassium salt (2517S), glucoarabin potassium salt (2516S), homoglucocamelinin potassium salt (2518), glucoraphanin potassium salt (2514S), glucocheirolin potassium salt (2524S), glucolepidiin potassium salt (2505S), glucoerucin potassium salt (2504S), glucoberteroin potassium salt (2501S), sinigrin potassium salt (7295S), gluconapin potassium salt (2507S), glucobrassicanapin potassium salt (2502S), progoitrin potassium salt (2515S) epiprogoitrin potassium salt (2512S), glucotropaeolin potassium salt (2510S), gluconasturtiin potassium salt (2508S), sinalbin potassium salt (2511S), glucolimnanthin potassium salt (2520S), glucobrassicin potassium salt (2520S), neoglucobrassicin potassium salt (2519S), 4-methoxyglucobrassicin potassium salt (2526), glucomorignin potassium salt (2506S), gallic acid (4993S), chlorogenic acid (4991S), ferulic acid (4753S), ellagic acid (6075), vanillin (6110S), caffeic acid (6034S), syringic acid (6011), p-coumaric acid (4751S), rosmarinic acid (4957S), 4-hydroxybenzoic acid (6099), protocatechuic acid (6050), 2’-hydroxyflavanone (1180), 7-hydroxyflavanone (1212), 4’-methoxyflavanone (1185), 5-methyxyflavanone (1186), apigenin-7-*O*-glucoside (1004S), luteolin-7-*O*-glucoside (1126S), isorhamnetin (120S), quercetin-3-*O*-rhamnoside (1236S), hyperoside (1027S), myricetin-3-*O*-galactoside (1355S), kaempferol-3-*O*-rutinoside (1053), ipriflavone (1328), naringin (1129S), were purchased from Extrasynthese (Lyon, France). Iberin (ab141944) and Sulforaphane (ab141969) were purchased from Abcam (Cambridge, UK. Allyl isothiocyanate (36682), benzyl isothiocyanate (89983), phenethyl isothiocyanate (68488) and indole-3-carbinol (I7256) were purchased from Sigma Aldrich (Vouros, Nicosia, Cyprus).

### 2.2 Plant material cultivation, processing and storage

Fresh watercress samples were kindly provided by the Watercress Company, Dorchester, Dorset, UK. The aerial part of watercress plants including flowers, lateral buds, petioles and stems were kept at -20°C until further use. Then, they were immersed in liquid nitrogen prior to being dehydrated in a freeze-drier (Alpha 1-4 LSC Basics, Christ) at -55°C, 0.05 mbar for 96 hrs. The dried parts were sprayed with liquid nitrogen and milled to fine powder using a domestic blender. The freeze-dried watercress powdered samples were stored at -80°C in a sealed bag protected from air, humidity and light until further use.

### 2.3 Extraction of polyphenolic compounds

The extraction of polyphenolic compounds was performed according to Kyriakou et al. (2022b). Briefly, one (1.0) gr of each of the examined watercress samples were extracted with exhaustive maceration at 80°C, with aqueous methanol 80% (*v/v*) for 48 hrs (Kyriakou et al., 2022b). The resulting mixture was filtered through a Whatman filter paper (pore size: 4.0-12 μm). The process was repeated twice. The combined methanolic solutions were lyophilized on a freeze-drier (Alpha 1-4 LSC Basics, Christ). The reconstructed (in 100% methanol) extracts, were filter (0.22 μm) (mixed cellulose esters, MCE) syringe and directly injected into UPLC-MS/MS for analysis.

### 2.4 Extraction of GLs

The extraction of GLs was accomplished according to Yu et al. (2022) with some modification. Briefly, one (1.0) gr of each watercress sample (either flowers or leaves or stems) were mixed with 100 mL aqueous methanol 70% (*v/v*). The resulting suspension was heated at 80°C for 30 mins and then it was sonicated for further 30 mins at room temperature (25°C). The resulting extract was centrifuged at 3000 x *g* for 20 min and 1 mL of the supernatant was diluted with UPLC grade water (1:10 dilution). The diluted solution was passed through a 0.22 μm (mixed cellulose esters, MCE) syringe filter, and directly analyzed *via* UPLC MS/MS.

### 2.5 Hydrolysis of GLs and extraction of ITCs

The hydrolysis of GLs was performed based on modified previously published procedure (Kyriakou et al., 2022b). Briefly, five (5.0) g of each watercress sample (flowers, leaves or stems) was dissolved in 350 mL phosphate buffer saline (PBS) (pH 7.0) containing 0.5 mmol of ascorbic acid. The formed suspension was heated at 37°C and stirred vigorously for 2 hrs. Then, the hydrolyzed mixture was extracted by stirring at 37°C for further 2 hrs with either 400 mL hexane (for PEITC extraction) or dichloromethane [for iberin (IBN), sulforaphane (SFN), indole-3-carbinol and benzyl isothiocyanate (BITC)] or diethyl ether for allyl isothiocyanate (AITC). Upon completion of the extraction, the organic phase was isolated, dried over magnesium sulfate and concentrated under reduced pressure at 40°C. The formed oils were reconstituted in acetonitrile, filtered twice through a 0.22 μm (mixed cellulose esters, MCE) membrane and rapidly mixed with 500 μL of 2M ammonia is isopropanol (apart from indole-3-carbinol enriched extract). The formed solutions were allowed at 25°C for 24 hrs. Then, the solvents were evaporated to dryness under reduced pressure. The dried thiourea derivatives were taken up in methanol containing 0.1% TFA.

### 2.6 Preparation of standards and samples

Stock solutions of 4-hydroxybenzoic acid, protocatechuic acid, gallic acid, vanillin, syringic acid, p-coumaric acid, caffeic acid, ferulic acid, rosmarinic acid, chlorogenic acid, ellagic acid, 7-hydroxyflavanone, 4’-methoxyflavanone, apigenin-7-*O*-glucoside, isorhamnetin, quercetin-3-*O*-rhamnoside, quercetin-3-*O*-rutinoside (rutin), naringin, kaempferol-3-*O*-rutinoside, hyperoside, myricetin-3-galactoside, were prepared in methanol. Luteolin-7-*O*-glucoside in acetonitrile/water mixture (1:1) and 2’-hydroxyflavanone, 5-methoxyflavanone and ipriflavone in methanol/acetonitrile mixture (1:1). Glucolepidiin, sinigrin, gluconapin, glucobrassicanapin, progoitrin, epiprogoitrin, glucotropaeolin, glucoerucin, gluconasturtiin, glucoiberin, glucoraphenin, glucoberteroin, glucoraphanine, glucocheirolin, glucobrassicin, neoglucobrassicin, 4-methoxyglucobrassicin, glucocamelinin, homoglucocamelinin, glucoarabin, glucomoringin, sinalbin and glucolimnanthin in 70% (*v/v*) methanol. All stock solutions were 1000 ppm.

For the content analysis of ITCs and indole-3-carbinol, the analytical standards were added with 2M ammonia in isopropanol for 24 hrs and then were evaporated to dryness under a nitrogen stream and reconstituted in a methanol: 0.1% (*v/v*) TFA mixture (4:1) whereas indole-3-carbinol was directly dissolved in methanol: 0.1% (*v/v*) TFA mixture (4:1) at a concentration of 1000 ppm. Working standard solution was made by diluting the individual standard stock solutions with ice cold methanol. Watercress extracts were diluted with ice cold methanol at final concentration of 25 ppb. Each solution was kept in the dark, protected from light in order to minimize the autooxidation of polyphenols. In addition, stock, standard and sample solutions were stored at -20°C before use. All prepared solutions were passed through 0.22 μm (mixed cellulose esters, MCE) membrane filtered prior UPLC-QqQ-ESI-MS/MS analysis.

### 2.7 Quantification of polyphenolic compounds, intact GLs and ITCs

#### 2.7.1 Liquid chromatography (LC) conditions

For the detection and quantification of the listed polyphenols, a Waters Acquity UPLC system (Waters Corp., Milford, MA, USA) equipped with an autosampler chamber, two pumps and a degasser, was used. The chromatographic separation was performed on an ACQUITY UPLC BEH C18 (100 x 2.1 mm, particle size: 1.7 μm) column (Waters Corp., Milford, MA, USA), heated at 30°C and eluted as it was previously reported with some modification for polyphenolic compounds, intact GLs and ITCs respectively (Song et al., 2005; Tian et al., 2005; Zhu et al., 2020).

##### 2.7.1.1 Polyphenolic compounds

Briefly, the mobile phase was consisted of a solution of acetonitrile (eluent A) and formic acid 0.1% (*v/v*) (eluent B). A flowrate of 0.3 mL/min was used and the linear gradient conditions applied consisted of 5-100% A (0-4 min), 100-90% A (4.0-4.1 min), 90%A (4.1-5 mins), 90-5% A (5-5.01 mins) and 5% A (5.1-6 mins).

##### 2.7.1.2 Intact GLs

The mobile phase consisted of methanol (eluent A) and formic acid 0.1% (*v/v*) (eluent B). A flowrate of 0.2 mL/min was used and the linear gradient conditions were 10% A (0-3 mins), 25% A (3-5 mins), 60% A (5-6 mins) and 0% A (6-6.2 mins), 10% A (6.2-9 mins).

##### 2.7.1.3 Derivatized ITCs and indole-3-carbinol

The mobile phase was consisted of a solution of 80% methanol (eluent A) and trifluoroacetic acid (TFA) 0.1% (*v/v*) (eluent B). A flowrate of 0.3 mL/min was used and the linear gradient conditions applied consisted of 50% A (0-5 mins), 60% A (5-10 mins), 70% A (10-20 mins) and 80% A (20-35 mins). The injection volume for all standards and analytes was 10 μL, and the autosampler temperature was set at 4°C during all analyses.

#### 2.7.2 MS/MS conditions

For the MS/MS experiments, a Xevo Triple Quadrupole (TQD) Mass detector (Waters Corp., Milford, MA, USA) was operated in either positive or negative ionization mode (ESI±). Detection of the various analytes was performed using selected ion recording (SIR) mode utilizing the collision voltage (MS1), individually determined for each analyte. Quantitative analysis was accomplished using selected multiple reaction monitoring (MRM) mode. The MRM conditions were optimized for each standard, by MS manual tuning of each standard prior to sample analysis at a concentration of 1 ppm (**Tables S1–S3**). In order to acquire maximum signals, the optimized tuning parameters were as follow: capillary voltage: 2.5-3.0 kV; cone voltage: 36 V; source temperature: 150°C; disolvation temperature: 500°C; source disolvating gas flow: 1000 L/h and gas flow: 20 L/h. High-purity nitrogen gas was used as the drying and nebulizing gas, whereas ultrahigh-purity argon was used as a collision gas. The data acquisition and processing were performed on MassLynx software (version 4.1).

### 2.8 Statistical analysis

All statistical analyses and Pearson’s correlation plotswere generated *via* R Statistical Software (2022), heatmaps were plotted using heatmap.2 from the gplots package in R. Statistical comparison of the analyte content among the aerial parts of watercress was performed by one-way ANOVA test, with post-hoc Tukey test for multiple comparisons utilizing appropriate software (GraphPad Prism version 8.0.1).

## 3 Results

**Figure 1:**
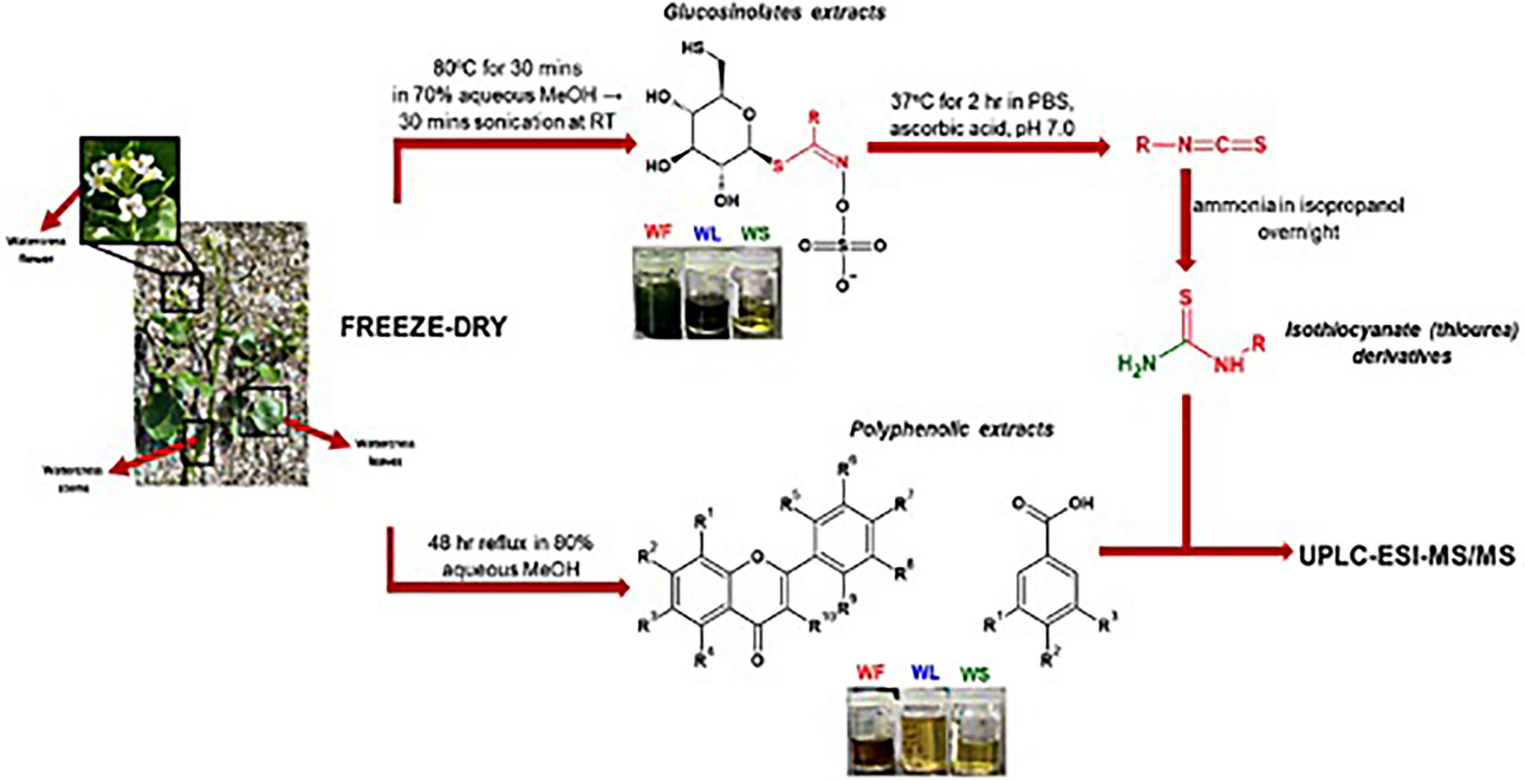
The methodological pipeline for the determination of polyphenolic and GL contents in the aerial parts of watercress: WF, watercress flowers; WL, watercress leaves; and WS, watercress stems.

The experimental set-up involved the segregation of flowers, leaves and stems followed by their dehydration and pulverization into a fine powder. Various extraction procedures were followed in order to obtain fractions of polyphenolic acids, GLs and ITCs (in the form of thiourea derivatives) (**Figure 1**).

### 3.1 Detection of GLs and polyphenolic compounds in aerial watercress parts

**Figure 2:**
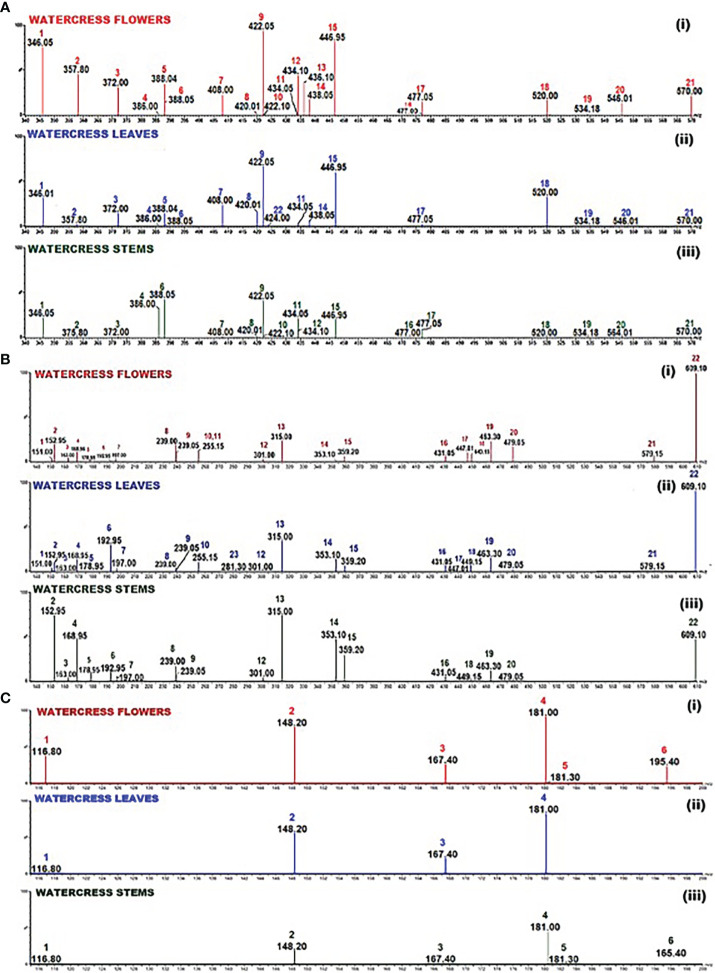
Selected Ion Recording (SIR) spectrum of: **(A)** the GL-enriched fractions of watercress (i) flowers, (ii) leaves and (iii) stems. Scanning (m/z; 340-570) was performed using collisions energies and m/z in the negative electrospray ionisation (ESI-) mode according to the collision energy as pointed in **Table S1**; 1 - glucolepidiin, 2 - sinigrin, 3 - gluconapin, 4 - glucobrassicanapin, 5 - progoitrin, 6 - epiprogoitrin, 7 - glucotropaeolin, 8 - glucoerucin, 9 - gluconasturtin, 10 - glucoiberin, 11 - glucoraphenin, 12 - glucoberteroin, 13 - glucoraphanine, 14 - glucocheirolin, 15 - glucobrassicin, 16 - neoglucobrassicin, 17 - 4-methoxyglucobrassicin, 18 - glucocamelinin, 19 - homoglucocamelinin, 20 - glucoarabin, 21 - glucomoringin, 22 – sinalbin; **(B)** the polyphenolic compounds in the hydromethanolic fraction of watercress flowers, leaves and stems. Scanning (m/z; 135-610) in both positive and negative electrospray ionisation (ESI±) mode according to the collision energy as pointed in **Table S2**; 1 - vanillin, 2 - protocatechuic acid, 3 - p-coumaric acid, 4 - gallic acid, 5 - caffeic acid, 6 - ferulic acid, 7 - syringic acid, 8 - 2’-hydroxyflavanone, 9 - 7-hydroxyflavanone, 10 - 4’-methoxyflavanone, 11 - 5’-methoxyflavanone, 12 - ellagic acid, 13 - isorhamnetin, 14 - chlorogenic acid, 15 - rosmarinic acid, 16 - kaempferol-3-O-rutinoside, 17 - quercetin-3-O-rhamnoside, 18 - luteolin-7-O-glucoside, 19 - hyperoside, 20 - myricetin-3-galactoside, 21 - naringin, 22 - quercetin-3-O-rutinoside, 23 – ipriflavone; **(C)** the derivatised into thiourea ITCs. Scanning (m/z 116.80-195.40) in positive electrospray ionisation mode according to the collision energy listed in **Table S3**; 1 - allyl thiourea, 2- indol-3-carbinol, 3 - benzyl thiourea, 4 - phenethyl thiourea, 5 - iberin thiourea, 6 - sulforaphane thiourea.

The first part of the analysis involved the detection of GLs with the prosthetic group been either sulfoxide or methyl sulphate or saturated aliphatic or methyl sulphite or allylic or aromatic. For this purpose, a selected ion recording (SIR) experiment was run involving watercress flower, leaves and stems in their GL-enriched extract in order to identify the masses of all tested GLs using the collision energy obtained from the manual tuning of each of the respective GL standards (**Figure 2A**; **S1**).

The scanning results revealed that among all 23 screened GLs, in the flowers, the levels of sinalbin and glucolimnanthin were below the detection limit of our method, in addition to those obtained from the leaves (e.g., glucoiberin, glucoberteroin, glucoraphanine, neoglucobrassicin and glucolimnanthin). Moreover, those obtained from the stems, levels of glucoberteroin, glucoraphanine, glucocheirolin, sinalbin and glucolimnanthin were not detected as well. In all three extract samples, gluconasturtiin and glucobrassin were at the highest abundance. Overall, the flower samples contained the highest abundance of GLs compared to leaves and stems. Finally, the same approach was followed for the polyphenolic (e.g., phenolic acids, flavanones, flavones, flavonols and isoflavone) compounds (**Figures 2B**; **S2**) as well as derivatised ITCs (thioureas)/indole-3-carbinol (**Figures 2C**; **S3**).

### 3.2 Standardization of UPLC and MS conditions

Afterwards, we sought into the quantification of each of the GLs individually by employing MRM transitions and by utilising commercially available reference standards. Finally, the combination of the mobile phase, elution mode, flow rate and column used for the separation were chosen in order to acquire the optimal signal for all the analytes.

For the determination of the optimum mobile phase several combinations were applied including methanol/water and acetonitrile/water in different ratios, with none of them being effective in the improvement of the shape of the peaks. Acidification of water with 0.1% (*v/v*) formic acid, was chosen since it allowed to obtain peaks with better symmetry and shape. Additionally, the presence of formic acid facilitated the ionization of the compounds. In the case of derivatized ITCs, TFA was preferred to rather than formic acid, as it was lower the pH of the mobile phase and improved the width of the peaks. Improvement of the shape of the peaks was achieved by increasing the column temperature to 30°C rather than 20°C for GLs and polyphenolic compounds and to 35°C for ITCs as it was previously suggested by others (Pilipczuk et al., 2017; Ruslin et al., 2022). For the ionization of polyphenols, the electrospray ionization (ESI) with either negative or positive (ESI^±^) mode was used while GLs and derivatized ITCs were ionized under ESI^-^ and ESI^+^ respectively.

### 3.3 Method validation

The analytical method was validated according to the guidelines of European Medicines Agency (European Medicine Agents, 1995). Namely, parameters including, linearity, limit of detection (LOD), limit of quantification (LOQ), precision, accuracy were determined (**Tables S4–6**). The generated calibration curves of the standards were plotted with linear regression equation of peak areas versus various concentrations ranging from 0.65 to 505.60 ppb for polyphenolic compounds (**Figure S4**), 1.95-250 ppm for GLs (**Figure S5**) and derivatized ITCs (**Figure S6**). All polyphenolic compounds and GLs demonstrated good linearity in the range of 0.81-513.20 ppb and1.50-258.20 ppb respectively whereas the correlation coefficients (R^2^) were >0.99 for all the analyzed standards (**Table 1**).

**Table 1:** Quantitative data demonstrating the screened glucosinolates, polyphenolic compounds and isothiocyanates in the aerial parts (flowers, leaves and stems) of watercress.

Analyte	Watercress flowers	Watercress leaves	Watercress stems
	ng of analyte/g of dry extract		
GLUCOSINOLATES			
Glucoiberin	0.37 ± 0.06^b^	N.D.	0.14 ± 0.04^a^
Glucoraphanine	17.98 ± 0.50^b^	N.D.	0.04 ± 0.01^a^
Glucocamelinin	5.97 ± 0.58^b^	10.44 ± 0.32^c^	0.040 ± 0.006^a^
Homoglucocamelinin	0.41 ± 0.03^b^	0.22 ± 0.03^a^	N.D.
Glucoarabin	6.20 ± 0.14	N.D.	N.D.
Glucoraphenine	0.48 ± 0.08^a^	3.57 ± 0.11^b^	4.12 ± 0.06^b^
Glucocheirolin	10.26 ± 0.96^b^	0.23 ± 0.03^a^	0.010 ± 0.005^a^
Glucolepidiin	67.78 ± 0.84^c^	8.30 ± 0.38^b^	3.53 ± 0.16^a^
Glucoberteroin	21.13 ± 0.92^b^	N.D.	0.65 ± 0.03^a^
Glucoerucin	0.19 ± 0.02^b^	3.15 ± 0.10^c^	0.020 ± 0.002^a^
Sinigrin	30.63 ± 0.37^c^	0.21 ± 0.02^a^	0.030 ± 0.002^a^
Gluconapin	14.95 ± 0.08^c^	3.24 ± 0.16^b^	0.0100 ± 0.0006^a^
Glucobrassicanapin	0.090 ± 0.001^b^	0.05 ± 0.01^a^	5.25 ± 0.09^c^
Epiprogoitrin	2.13 ± 0.08^c^	0.34 ± 0.03^a^	7.48 ± 0.05^b^
Progoitrin	15.38 ± 0.47^b^	3.96 ± 0.47^a^	N.D.
Glucolimnanthin	N.D.	N.D.	N.D.
Sinalbin	N.D.	0.02 ± 0.01	N.D.
Gluconasturtiin	82.11 ± 0.63^c^	36.25 ± 0.74^b^	9.20 ± 0.11^a^
Glucotropaeolin	9.59 ± 0.48^c^	6.22 ± 0.04^b^	0.0200 ± 0.0008^a^
Glucobrassicin	74.93 ± 0.85^c^	21.11 ± 0.10^b^	1.61 ± 0.24^a^
Glucomoringin	9.42 ± 0.12^b^	0.020 ± 0.001^a^	0.10 ± 0.14^a^
4-methoxyglucobrassicin	7.76 ± 0.77^c^	2.29 ± 0.13^b^	0.95 ± 0.03^a^
Neoglucobrassicin	0.48 ± 0.02^a^	N.D.	0.45 ± 0.07^a^
4-hydroxybenzoic acid	N.D.	N.D.	N.D.
Protocatechuic acid	134.72 ± 1.21^c^	96.79 ± 0.79^b^	73.59 ± 0.48^a^
Gallic acid	60.74 ± 0.44^c^	56.04 ± 1.93^b^	32.25 ± 0.26^a^
Vanillin	11.20 ± 0.92^b^	3.60 ± 0.07^a^	N.D.
Syringic acid	13.83 ± 0.09^c^	3.94 ± 0.06^b^	1.59 ± 0.07^a^
p-coumaric acid	17.30 ± 0.40^b^	22.52 ± 4.22^c^	3.58 ± 0.06^a^
Caffeic acid	30.89 ± 0.12^c^	13.44 ± 0.35^b^	7.63 ± 0.07^a^
Ferulic acid	17.70 ± 0.38^b^	306.98 ± 2.44^c^	7.94 ± 0.07^a^
Rosmarinic acid	33.65 ± 1.01^c^	26.54 ± 0.41^b^	24.77 ± 0.19^a^
Chlorogenic acid	41.34 ± 1.00^c^	59.34 ± 0.17^b^	38.84 ± 0.43^a^
Ellagic acid	6.51 ± 1.06^c^	2.38 ± 0.23^b^	0.65 ± 0.04^a^
2’-hydroxyflavanone	49.47 ± 0.71^c^	32.75 ± 0.78^b^	12.71 ± 0.30^a^
7-hydroxyflavanone	39.94 ± 0.67^c^	19.33 ± 0.53^b^	7.31 ± 1.15^a^
4’-methoxyflavanone	12.96 ± 0.81^b^	6.12 ± 0.10^a^	N.D.
5-methoxyflavanone	6.15 ± 0.02	N.D.	N.D.
Apigenin-7-*O*-glucoside	15.64 ± 0.24^b^	3.29 ± 0.03^a^	N.D.
Luteolin-7-*O*-glucoside	35.18 ± 1.14^c^	7.39 ± 0.09^b^	1.00 ± 0.07^a^
Isorhamnetin	289.40 ± 1.37^c^	196.47 ± 3.65^b^	65.67 ± 0.81^a^
Quercetin-3-*O*-rhamnoside	35.08 ± 0.70^b^	2.22 ± 0.24^a^	N.D.
Quercetin-3-*O*-rutinoside	1459.30 ± 12.95^c^	1197.86 ± 4.24^b^	41.02 ± 0.18^a^
Hyperoside	125.73 ± 1.75^c^	87.96 ± 0.53^b^	6.51 ± 0.26^a^
Myricetin-3-galactoside	63.15 ± 1.94^c^	12.46 ± 0.33^b^	0.81 ± 0.04^a^
Kaempferol-3-*O*-rutinoside	257.54 ± 2.31^c^	10.41 ± 0.52^b^	2.25 ± 0.13^a^
Ipriflavone	N.D.	N.D.	N.D.
Naringin	21.72 ± 0.39^b^	11.16 ± 0.11^a^	N.D.
Sulforaphane	9.43 ± 0.24^b^	N.D.	0.16 ± 0.01^a^
Iberin	0.49 ± 0.01^b^	N.D.	0.060 ± 0.007^a^
Benzyl isothiocyanate	13.32 ± 0.39^c^	0.77 ± 0.05^b^	0.020 ± 0.006^a^
Phenethyl isothiocyanate	273.89 ± 0.88^c^	125.02 ± 0.52^b^	64.70 ± 0.90^a^
Allyl isothiocyanate	16.04 ± 0.81^c^	0.75 ± 0.13^b^	0.040 ± 0.004^a^
Indole-3-carbinol	191.44 ± 1.99^c^	95.55 ± 0.94^b^	16.64 ± 0.28^a^

N.D. indicates the non – detected metabolites. Data are the means of six independent experiments ± SD. Means ± SD followed by distinct letters in the same row statistically differ according to Tukey’s *post hoc* test, (*p*< 0.05).

Finally, we evaluate the reproducibility of the UPLC-QqQ-ESI-MS/MS method by means of determining the % of recovery (**Tables S4–S6**) based on the quantification protocol followed for GLs, polyphenolic compounds and derivatized ITCs respectively. For this purpose, each watercress extract was spiked with mixtures of standard solutions of various GLs or polyphenols or ITCs. Spike samples were prepared in triplicates and the results were of at least six repetitions. The % recovery was calculated according to equation (**1**), where A is the final amount detected, A_0_ is the initial amount and A_a_ is the added amount:


(**1**) $$\text{\% recovery}=\left[\frac{\text{A}-{\text{A}}_{0}}{{\text{A}}_{\text{a}}}\right]\text{ x }100\%$$

The average recovery of all of the polyphenolic compounds ranged between 89.2% and 102.6% while the respective range for all intact GLs was 84.0-101.2%. In the case of derivatized ITCs (thioureas) the recovery ranged between 80.2-96.7% thereby demonstrating the accuracy and reproducibility of our methodological approach.

### 3.4 Linearity, accuracy and precision of the methodology

Limit of detection (LOD) and quantification (LOQ) values were calculated based on the signal to noise ratio (*S/N*) which was set at 3 and 10 respectively. The range of LOD and LOQ determination for polyphenolic compounds were 0.65-109.90 ppb and 1.21-105.20 ppb respectively, whereas for intact GLs was 0.27-6.36 and 0.79-22.65 ppb. Furthermore, the calculated LOD and LOQ ranges for the various thioureas (which correspond to ITCs) were between 2.73-24.43 ppb and 9.12-81.45 ppb respectively. Based on the values presentenced in **Table S5**, from all the polyphenols, the LOD of *p*-coumaric acid was the lowest and that of gallic acid was the highest suggesting that the sensitivity of *p*-coumaric acid was better than for the other polyphenols ionized in ESI^-^. On the contrary, the highest sensitivity was shown to be for luteolin-7-*O*-glucoside and 7-hydroxyflavanone compared to the others being ionized in ESI^+^. Furthermore, the LOD for glucoiberin was the lowest whereas the highest LOD was denoted for epiprogoitrin thus indicating a higher sensitivity in detecting glucoiberin among all other GLs (**Table S4**). Moreover, the LOD of indole-3-carbinol was the highest whereas the respective one for AITC was the lowest suggesting better sensitivity among the thioureas ionized *via* ESI^+^ (**Table S6**).

Finally, the degree of similarity of multiple samples within the same family (bearing the same functional groups) was evaluated by means of percent relative standard deviation (% RSD). In order determine the % RSD, six replicated samples, at the same concentration, were analyzed within one day for intra-day precision and within five continuous days for inter-day precision. More specifically, % RSD results of both intra- and inter-day were 1.89-4.6% (for intact GLs) (**Table S4**) 0.5-4.2% (for polyphenolic compounds) (**Table S5**), and 1.04-4.02% (**Table S6**) (for derivatized ITCs), respectively.

### 3.5 Polyphenolic compounds and intact GLs fragmentation

The collision gas fragmentation of the precursor molecules led to the formation of increasingly lower m/z (fragments) ions at *m/z* 93-197. In particular, the fragmentation pattern of the phenolic acids [e.g., 4-hydroxybenzoic acid (136.95>93), protocatechuic acid (*m/z* 152.9>108.95), gallic acid (*m/z* 68.95>124.95), *p*-coumaric acid (*m/z* 163>119) and caffeic acid (*m/z* 178.95>134.95)] is characterized by loss of the carboxyl group (*m/z* 44) [M-H-C(=O)OH]^-^. On the other hand, in some phenolic acids [e.g., vanillin (*m/z* 151>136), syringic acid (*m/z* 197>182)], anionic species were produced by the loss of their methyl group; [M-H-CH_3_]^-^ whereas in the case of ferulic acid (*m/z* 192.95>178) a loss of both methyl and carboxyl acid moieties occurred [M-H-59]^-^. The gas-induced fragmentation of chlorogenic acid resulted in loss of the caffeyl functionality [M-H-caffeyl]^-^ yielding *m/z* 359.2>145. Whereas, the fragment produced from rosmarinic acid was generated upon dehydration of the cleaved ester products [M-H-179]^–^H_2_O or [M-H-197]^–^2H_2_O, *m/z* 359.2>161. Since chlorogenic and rosmarinic acid share the same base peak, their identification was performed based on the fragmentation pattern, which included fragments in the range of 65-173 *m/z*. The quantification of each acid was then performed on the most abundant fragment of each compound, different from the base peak one.

Similar observations were also noted in flavanones. Namely, 2’-hydroxyflavanone, 7-hydroxyflavanone, apigenin-7-*O*-glucoside, isorhamnetin, quercetin-3-*O*-rutinoside, quercetin-3-*O*-rhamnoside, hyperoside, myricetin-3-galactoside, kaempferol-3-*O*-rutinoside and nariginin produced [M-H]^-^ at *m/z* 239-609.1 whereas 4’-methoxyflavanone, 5’-methoxyflavanone, luteolin-7-*O*-glucoside, and ipriflavone produced [M+H]^+^ at *m/z* 255.15-449.15. On the other hand, MS^2^ fragmentation generated fragment ions with signals at *m/z* 91.15-300 and *m/z* 151.3-287.1 for negative and positive ionization modes respectively. Namely, collision-induced fragmentation of 2’-hydroxyflavanone resulted in the loss of chromone [M-H-chromone]^-^, whereas fragmentation of 7-hydroxyflavanone resulted in the loss of chromenone [M-H-chromenone]^-^ thus generating signals *m/z* 240.47>93.1 and *m/z* 240>91.15, respectively. In the case of 4’-methoxyflavanone (*m/z* 255.15>240) cleavage of the methyl group (*m/z* 15) was noticed. Furthermore, during MS^2^ fragmentation, apigenin-7-*O*-glucoside (*m/z* 431.15>268.35), luteolin-7-*O*-glucoside (*m/z* 449.15>187.1) and hyperoside (*m/z* 463.3>300) yielded aglucon fragments by the loss of glucoside [M ± H-glucoside]^±^. In respect to quercetin-3-*O*-rutinoside and quercetin-3-*O* rhamnoside, the collision-induced fragmentation generated signals [M-2H-rutinoside]^2-^ at *m/z* 447.01>300 and [M-H-rhamnoside]^-^ at *m/z* 609.1>300, respectively. A similar fragmentation pattern was also noticed in the case of kaempferol-3-*O*-rutinoside and naringin as the parental molecules lost rutinoside and glucorhamnoside respectively thus allowing the detection of the fragment ionsat *m/z* 431.05>255.3 and *m/z* 579.15>271.1. Finally, fragmentation of isorhamnetin led to elimination of 2,4,6-trihydroxybenzaldehyde [M-H-164]^-^ thus generating a signal at *m/z* 315>151 whereas elimination of isopropyl moiety of ipriflavone [M-2H-*i*Pr]^2-^ yielded a signal at *m/z* 281.3>240.

With respect to the MS^2^ fragmentation of GLs, it appears that all of them follow the same fragmentation pattern. Collision gas fragmentation of the precursor GLs led to the production of a maximum abundant precursor ion at *m/z* 97. Therefore, the MS^2^ ionization induced a cleavage at the sulfate functionality forming an ion [SO_3_H^-^] with *m/z* 97. Glucomoringin is the only GL with a distinct MS^2^ fragmentation pattern and which produced a strong signal at *m/z* 570.

In general, ITCs can be detected in the ESI^-^ mode, however, the analysis is of low sensitivity thus preventing the accurate quantification of the ITC content. Therefore, ITCs were derivatized into their respective thioureas upon treatment with alcoholic solution of ammonia. This modification increased the sensitivity and accuracy of the method and thus facilitating their quantification (Song et al., 2005). In addition, in this study, we utilized indole-3-carbinol as a representative hydrolysis product of the elevated glucobrassicin. This is because, the respective indolyl-3-methyl ITC is unstable in the aqueous environment of hydrolysis and it is rapidly rearranged into the respective indolyl alcohol (Błoch-Mechkour et al., 2010). The fragmentation of all derivatized ITCs followed the same pattern as the MS^2^ fragmentation promoted the loss of ammonia (*m/z* 18) thereby resulting in signals for allyl thiourea *m/z* 99.8, iberin thiourea *m/z* 163.4, benzyl thiourea *m/z* 148.9 and sulforaphane thiourea *m/z* 177.2. In the case of phenethyl thiourea the collision energy fragmentation led to the loss of thiourea [M-thiourea] ^+^ (*m/z* 76) allowing the detection of the transition *m/z* 181.0>104.99. Finally, fragmentation of indole-3-carbinol resulted in the loss of the methanol (*m/z* 32) [M-MeOH]^+^ thus generating a transition at *m/z* 148.0>117.2.

### 3.6 Quantification of intact GLs, polyphenolic compounds and ITCs

**Figure 3:**
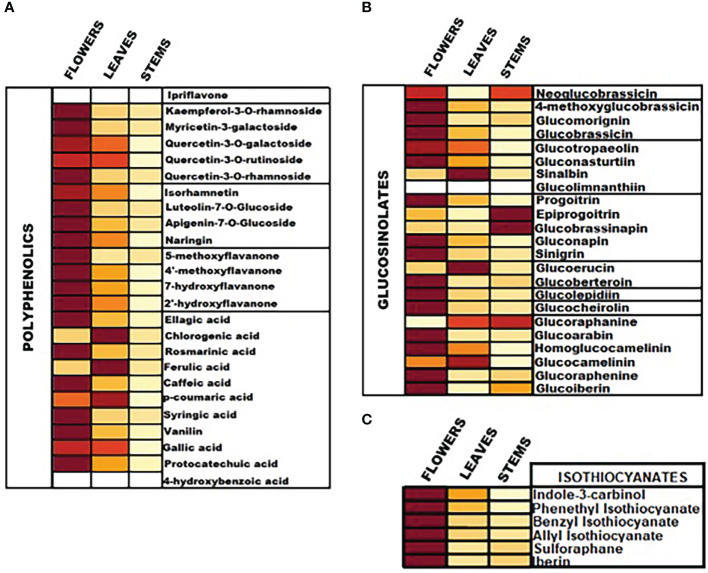
Heatmap representation of the relative content of **(A)** polyphenolic compounds, **(B)** intact GLs and **(C)** ITCs in the aerial parts of watercress including flowers, leaves and stems, upon LC-MS/MS quantification.

The content of intact GLs, polyphenolic compounds and ITCs was assessed by means of both qualification and quantification. Absolute quantities of individual analytes are reported in **Table 1** and representation of the total content of the analytes in the aerial parts is illustrated in the heatmap as part of **Figure 3**. Overall, our findings suggest that among all screened GLs, watercress flowers lack of two aromatic GLs namely glucolimnanthin and sinalbin. In addition, glucoiberin, glucoraphanin, glucoarabin, glucoberteroin, glucolimnanthin and neoglubrassicin were not present in watercress leaves. Finally, homoglucocamelinin, glucoarabin, progoitrin, glucolimnanthin and sinalbin were not detected in watercress stems as well.

Overall, the highest content of GLs was detected in watercress flowers over watercress leaves and stems as proved by statistical analysis. The next aerial part with the highest contest was leaves followed by stems. Among all screened GLs, gluconasturtiin was the most dominant GL in all aerial components of watercress [82.11 ± 0.63 ng/g of dry extract – for watercress flowers; 36.25 ± 0.74 ng/g of dry extract – for watercress leaves; and 9.20 ± 0.11 ng/g of dry extract for watercress stems]. Interestingly, on watercress flowers, glucolepidiin (67.78 ± 0.84 ng/g of dry extract) and glucobrassicin (74.93 ± 0.85 ng/g of dry extract) were also found in high abundance. On the contrary, in watercress leaves, increased glucocamelinin (10.44 ± 0.32 ng/g of dry extract) was observed whereas on watercress stems, glucoraphenin (4.12 ± 0.06 ng/g of dry extract), glucobrassicanapin (5.25 ± 0.09 ng/g of dry extract) and epiprogoitrin (7.48 ± 0.05 ng/g of dry extract) were in the highest proportion. The difference in the content of glucoraphanine, glucocheirolin, glucoberteroin, sinigrin and glucomorignin in watercress leaves and stems, respectively, were not of statistical significance. Similar observations were also noted in the content of sinalbin and neoglucobrassicin as there wasn’t any statistical alteration of their content between watercress flowers and stems. The highest levels of glucobrassinapin were observed in watercress stems, whereas there wasn’t any significant difference in the respective levels of watercress flowers and stems.

In addition, the content of polyphenolic compounds (phenolic acids and flavonoids) was determined in the aerial parts of watercress. The compounds with the highest abundance, in watercress flowers, were quercetin-3-*O*-rutinoside (1459.30 ± 12.95 ng/g of dry extract), isorhamnetin (289.40 ± 1.37 μng/g of dry extract) and kaempferol-3-*O*-rutinoside (257.54 ± 2.31 ng/g of dry extract) The difference in the detected levels of the above-mentioned metabolites was of statistical significance when compared with the respective levels of watercress leaves and stems. The same pattern was followed in watercress leaves, however, sharp elevated levels of ferulic acid (306.98 ± 2.44 ng/g of dry extract) (p<0.0001 between watercress flowers-leaves and stems-leaves) were also recorded. For watercress stems, the highest concentration of polyphenolic compounds was determined in protocatechuic acid (73.59 ± 0.48 ng/g of dry extract), chlorogenic acid (38.84 ± 0.43 ng/g of dry extract) and isorhamnetin (65.67 ± 0.81 ng/g of dry extract). However, those levels were scientifically lower when compared to watercress flowers and stems.

On another note, we determined that in all aerial parts of watercress tested, the PEITC content was of the highest abundance followed by indole-3-carbinol. Between the three aerial parts, the highest accumulation of PEITC and indole-3-carbinol was noted on flowers (273.89 ± 0.88 and 191.44 ± 1.99 ng/g of dry extract respectively) (p<0.0001) while the lowest one on stems (64.70 ± 0.90 and 16.64 ± 0.28 ng/g of dry extract respectively). Interestingly, our results shown that SFN and IBN while being present in watercress flower and leaves samples this was not the case in watercress stems, with the different in their content in watercress leaves and stems been non-statistical significance

### 3.7 Correlation of intact GLs and ITCs

**Figure 4:**
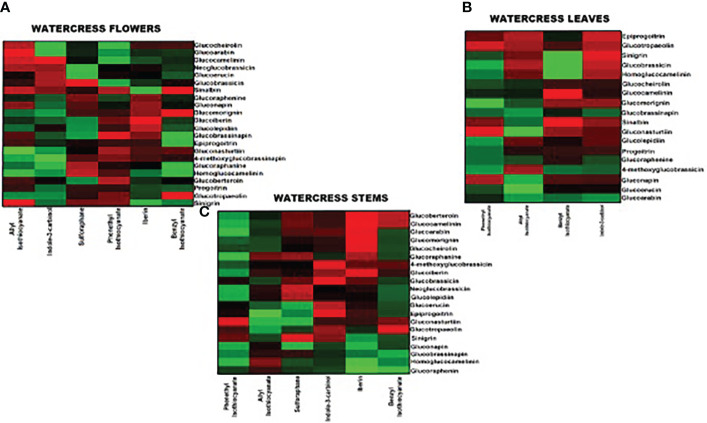
Pearson’s correlation analysis between intact GL and ITC contents on watercress **(A)** flowers, **(B)** leaves and **(C)** stems. Red indicates a positive correlation (+1.0) whereas bright and dark green denote no correlation or negative correlation (−1.0), respectively.

We performed a Pearson’s correlation analysis in order to observed any linear relationship between the GL and ITC contents in the aerial parts of watercress (**Figure 4**). Our results demonstrate a strong correlation between the precursors GLs and their direct hydrolysis by-products (e.g., sinigrin – AITC, glucoraphane – SFN, glucoiberin – IBN, glucotropaeolin – BITC, gluconasturtiin – PEITC and glucobrassicin – indole-3-carbinol). In addition, a strong correlation between each ITC and the general class of each GL was noted suggesting a potential contribution of more than one GL in the biosynthesis of specific ITCs. On the contrary, there is no correlation between GLs and ITCs of other classes. The same trends are followed in all aerial parts of watercress. On the other hand, Spearman’s correlation analysis (**Figure S7**) points that there is a correlation between GLs and ITCs of other classes. More specifically, it appears that allylic and indolyl ITCs are strongly correlated with aliphatic, sulfides and methyl sulfoxide-based GLs. This patterns also seems to be appeared in watercress flowers (**Figure S7A**) and watercress stems (**Figure S7C**). In addition to this, strong correlation also appears between aromatic and indolyl based ITCs and aliphatic, and allylic based GLs (**Figures S7B, C**).

## 4 Discussion

Quantitative determination of the various classes of GLs (e.g., sulfoxides, methyl sulphates, saturated aliphatics, methyl sulfites, allylics, aromatic and indolyls) and polyphenolic compounds (e.g., phenolic acids, flavanones, flavones, isoflavones and flavanols) in the aerial parts of watercress (e.g., flowers, leaves and stems) was performed by the detection of the corresponding masses of intact GLs and polyphenolic compounds utilizing SIR acquisition mode. On the other hand, their quantification was based on the employment of the MRM mode, which is characterized by sensitivity and selectivity. The co-elution of several GLs (e.g., glucoraphanine, glucoraphenine, glucoiberin, quercetin-3-*O*-rhamnoside and apigenine-7-*O*-glucoside) was observed during the UPLC-MS/MS analysis however, successful separation with MRM detection by-passed the potential effect of a simultaneous quantification. In addition, the MS/MS technique reduced the need for chromatographic resolution of individual analytes, minimizing the analytical time thus allowing a higher sample throughput.

GLs are highly polar metabolites, particularly due to the presence of thioglucosyl which behaves as a strong acid (Andini et al., 2020). Hence, the desulfation process was thought to be an essential step for their detection and quantification (Bennett et al., 2002; Förster et al., 2015). However, this methodology has many limitations including the self-degradation, self-dimerization and degradation. In addition, others have attempted to quantify GLs, by utilizing gas chromatography approaches, however, it was found that GLs are thermally unstable and so gas chromatography was withdrawn (Śmiechowska et al., 2010; Chaudhary et al., 2012; Wu et al., 2021). On the other hand, our approach is characterized by the considerably lower time of sample preparation, faster chromatographic separation and also the stability of GLs is not affected by the analysis.

In addition, many studies have been performed in order to quantify ITCs by means of utilizing the cyclo-condensation assay as well as liquid chromatography coupled to UV detector (LC/MS-UV) methodologies. However, these techniques are often accompanied with difficulties in the separation and identification of individual ITCs. On the other hand, the increased volatility of ITCs prevents them from gas chromatography analysis as they rapidly degraded (Hanschen et al., 2015; Pilipczuk et al., 2017). Consequently, derivatization methodologies have been employed based on the electrophilic center of the ITC moiety being used for further derivatization (e.g., *N-*acetyl cysteine bridging or glutathione bridging or mercaptoethanol) leading to the formation of mercapturic acid pathway intermediates which can be easily ionized with ESI and thus facilitate their quantification (Agerbirk et al., 2015; Pilipczuk et al., 2017; Nacca et al., 2021). In our study, we have chosen derivation with ammonia in order to form stable thioureas. The ammonia derivatization of ITCs is a clean fast and almost quantitatively approach which can be followed for the quantification of individual ITCs (Ji and Morris, 2003; Agerbirk et al., 2015). Our results are in agreement with Jeon et al. (2017) showing that the highest content of all accumulated ITCs was that of gluconasturtiin (Jeon et al., 2017). Moreover, the same group indicated that watercress flowers had the highest content of ITCs among all other plant parts examined. However, our data also shown the presence of increased levels of quercetin-3-rutinoside, isorhamnetin and kaempferol-3-*O*-rutinoside in watercress flowers. Overall, our data are in agreement with the work of others although they are lacking data regarding the localization of these metabolites (Zeb, 2015; Ma et al., 2021; Panahi Kokhdan et al., 2021). To the best of our knowledge, this is the first report which provides a comprehensive analysis of the content of a library of GLs, ITCs, phenolic acids and flavonoids in the aerial parts of watercress.

Finally, according to our data, watercress flowers are the most concentrated source of phenolic acids, flavonoids, GLs and ITCs followed by those of leaves and stems. The elevated quantities of polyphenols (flavonoids) and volatiles (including ITCs) in flowers can be attributed to the fact that they contain the reproductive organs which makes them more valuable for plant fitness (Strauss et al., 2004; Schiestl, 2014). Also, their role defers from that of leaves and stems despite the fact that they share similar chemistry (Kessler et al., 2013). For instance, it has been suggested that the existence of ITCs does not function entirely as a defense mechanism but also to attract pollinator signals related to plant reproduction (Bouwmeester et al., 2019). On the contrary, the existence of volatiles and phytochemicals on leaves and plants are primarily responsible for the activation of the plant defense mechanism upon herbivore of parasites attack (Dixon and Shaw, 2011; Palliyaguru et al., 2018).

Overall, throughout this report, we suggest a strong correlation between ITCs with their respective GLs within the same group. Pearson’s association analysis reveals that bioconversion of GLs into ITCs might be regulated either by the rearrangement of other ITCs or by the contribution of other GLs (which belong to the same group) into ITCs biosynthesis, depending on plant necessities. This report provides substantial evidence for the potential exploitation of various aerial parts of the watercress plant in order to enhance its capacity towards the development of pharmaceutical applications. Although mainly in watercress flowers and leaves (edible parts) there appear to be a plethora of nutrients and phytochemicals, stems (non-edible part) also provides sources of these metabolites, thereby makes it a promising source of waste recycling and valorization in the context of circular economy.

## Data availability statement

The original contributions presented in the study are included in the article/**Supplementary Material**. Further inquiries can be directed to the corresponding author.

## Author contributions

Conceptualization, SK, MP. Methodology, SK, KM, MP. Software, SK. Formal analysis, SK, KM, MP. Investigation, SK. Resources, TA, KS, PW, MP. Data curation, SK. Writing - original draft preparation, SK, MP. Writing - review and editing, SK, TA, KS, PW, DT, RF, AP, MP. Supervision, MP. Project administration, MP. Funding acquisition, MP. All authors contributed to the article and approved the submitted version.

## Funding

This work was supported by a grant provided by the Cyprus Institute of Neurology and Genetics (Telethon Cyprus), Nicosia, Cyprus (MIP).

## Conflict of interest

Author TA is employed by The Watercress Company, authors KS and PW are co-founders of Watercress Research Limited.

The remaining authors declare that the research was conducted in the absence of any commercial or financial relationships that could be construed as a potential conflict of interest.

## Publisher’s note

All claims expressed in this article are solely those of the authors and do not necessarily represent those of their affiliated organizations, or those of the publisher, the editors and the reviewers. Any product that may be evaluated in this article, or claim that may be made by its manufacturer, is not guaranteed or endorsed by the publisher.
