# Supplementary material for: Polyphenolics, glucosinolates and isothiocyanates profiling of aerial parts of Nasturtium officinale (Watercress)
Source: Front Plant Sci. 2022 Nov 15;13:998755. doi: 10.3389/fpls.2022.998755 (PMC9706002; doi:10.3389/fpls.2022.998755)
Supplement: Supplementary file 1 [file DataSheet_1.pdf]

Supplementary Material

Polyphenolic and glucosinolate profile distribution among the aerial parts of *Nasturtium officinale* (Watercress)

Sotiris Kyriakou[1], Kyriaki Mihailidou[1], Tom Amery[2], Paul Kyle[3], Paul G. Winyard[4], Dimitrios T. Trafalis[5], Franco Rodrigo[5,6], Aglaia Pappa[7], Mihalis I Panayiotidis[1*]

[1]Department of Cancer Genetics, Therapeutics & Ultrastructural Pathology, The Cyprus Institute of Neurology & Genetics, Nicosia 2371, Cyprus

[2]The Watercress Company, Dorchester DT2 8QY, UK

[3]Watercress Research Limited, Devon TQ12 4AA, UK

[4]Laboratory of Pharmacology, Medical School, National & Kapodistrian University of Athens, 11527 Athens, Greece

[5]Redox Biology Centre, University of Nebraska-Lincoln, Lincoln, NE 68583, USA

[6]Department of Veterinary Medicine & Biomedical Sciences, University of Nebraska-Lincoln, Lincoln, NE 68583, USA

[7]Department of Molecular Biology & Genetics, Democritus University of Thrace, 68100 Alexandroupolis, Greece

* Correspondence:
Mihalis I. Panagiotidis
mihalisp@cing.ac.cy

Table S1: Multiple Reaction Monitoring conditions for glucosinolates in UPLC-MS/MS analysis

Glucosinolate	Chemical formula	Molecular weight	[M-H]- (m/z)	MS^2 fragments (m/z)	Cone voltage (V)	Collision energy (eV)	Retention time (R_t)
Glucoiberin	$C_{11}H_{21}NO_{10}S_3$	423.48	422.1	97.2<br>259.15	33	26<br>25	1.22
Glucoraphanine	$C_{12}H_{23}NO_{10}S_3$	437.51	436.1	96.95<br>372.0	26	23<br>20	1.20
Glucocamelinin	$C_{18}H_{35}NO_{10}S_3$	521.66	520.0	97.0<br>456.4	26	26<br>19	5.88
Glucoarabin	$C_{17}H_{33}NO_{10}S_3$	547.64	546.01	331.05<br>96.95	35	26<br>32	7.42
Homoglucocamelinin	$C_{19}H_{37}NO_{10}S_3$	535.69	534.15	470.0<br>519.4	19	25<br>25	6.30
Glucoraphenin	$C_{12}H_{21}NO_{10}S_3$	435.59	434.05	96.95<br>259.0	32	16<br>18	1.21
Glucocheirolin	$C_{11}H_{21}NO_{11}S_3$	439.48	438.05	96.95<br>75.1	19	29<br>26	1.23
Glucolepidiin	$C_9H_{17}NO_9S_2$	347.36	346.05	75.1<br>96.95	26	26<br>14	1.27
Glucoerucin	$C_{12}H_{23}NO_9S_3$	421.51	420.01	96.9<br>259.0	32	19<br>19	3.75
Glucoberteroin	$C_{13}H_{24}NO_9S_3$	435.52	434.1	96.9<br>259.0	17	17<br>22	4.90
Sinigrin	$C_{10}H_{17}NO_9S_2$	359.37	357.8	96.95<br>194.93	35	17<br>21	1.44
Gluconapin	$C_{11}H_{19}NO_9S_2$	373.40	372.0	96.9	35	34	2.11
Glucobrassicanapin	$C_{12}H_{21}NO_9S_2$	387.43	386.0	96.95	23	12	3.65
Progoitrin	$C_{11}H_{19}NO_{10}S_2$	389.4	388.05	96.95<br>195.05	9	15<br>20	1.23
Epiprogoitrin	$C_{11}H_{19}NO_{10}S_2$	389.4	388.05	97<br>195.1	22	19<br>19	1.45

Glucotropaeolin	$C_{14}H_{19}NO_9S_2$	409.43	408.0	96.95<br>166.01	20	20<br>19	3.70
Gluconasturtiin	$C_{15}H_{21}NO_9S_2$	423.46	422.05	96.9	18	16	4.87
Sinalbin	$C_{14}H_{19}NO_{10}S_2$	425.43	424.0	75.15<br>96.95	20	26<br>21	1.71
Glucolimnanthin	$C_{15}H_{21}NO_{10}S_2$	439.46	438.05	96.95<br>260.0	22	22<br>18	4.39
Glucobrassicin	$C_{16}H_{20}N_2O_9S_2$	448.47	446.95	97.0<br>259.0	22	18<br>22	4.21
Neoglucobrassicin	$C_{17}H_{22}N_2O_{10}S_2$	478.49	477.0	97.05<br>446.0	22	22<br>11	5.64
4-methoxyglucobrassicin	$C_{17}H_{22}N_2O_{10}S_2$	478.49	477.05	195.0<br>275.0	22	20<br>20	5.17
Glucomoringin	$C_{20}H_{29}NO_{14}S_2$	571.57	570.0	570.0	28	10	1.28

Table S2: Multiple Reaction Monitoring conditions for polyphenolic compounds in UPLC-MS/MS analysis

Polyphenolic compound	Chemical formula	Molecular weight	[M-H]± (m/z)	MS² fragments (m/z)	Cone voltage (V)	Collision energy (eV)	Retention time (R_t)
4-hydroxybenzoic acid	$C_7H_6O_3$	138.12	136.95	65.0<br>93.0	23	25<br>13	1.88
Protocatechuic acid	$C_7H_6O_4$	154.12	152.95	108.95	25	13	1.64
Gallic acid	$C_7H_6O_5$	170.12	168.95	78.98<br>124.95	23	22<br>15	1.37
Vanillin	$C_8H_8O_3$	152.15	151.0	92.2<br>136.0	22	20<br>15	2.23
Syringic acid	$C_9H_{10}O_5$	198.17	197.0	122.95<br>182.0	27	23<br>13	1.93
p-coumaric acid	$C_9H_8O_3$	164.16	163.0	119.0	15	13	2.13
Caffeic acid	$C_9H_8O_4$	180.16	178.95	134.95	25	13	1.89
Ferulic acid	$C_{10}H_{10}O_4$	194.18	192.95	134.0<br>178.0	26	25<br>12	2.20
Rosmarinic acid	$C_{18}H_{16}O$	360.32	359.2	161.0<br>197.0	10	15<br>15	2.26
Chlorogenic acid	$C_{16}H_{18}O_9$	354.31	353.1	84.0<br>191.02	22	44<br>14	1.70
Ellagic acid	$C_{14}H_6O_8$	302.19	301	145.0<br>173.0	35	34<br>36	2.00
2’-hydroxyflavanone	$C_{15}H_{12}O_3$	240.27	239	119.3<br>93.1	40	25<br>16	3.42
7-hydroxyflavanone	$C_{15}H_{12}O_3$	240.27	239.05	135.2<br>91.15	41	25<br>23	3.42
4’-methoxyflavanone	$C_{16}H_{14}O_3$	254.29	255.15	240<br>161.3	31	17<br>22	3.78
5-methoxyflavanone	$C_{16}H_{14}O_3$	254.29	255.15	151.3	34	22	3.49
Apigenin-7-O-glucoside	$C_{21}H_{20}O_{10}$	432.38	431.15	268.35	35	22	2.15
Luteolin-7-O-glucoside	$C_{21}H_{20}O_{11}$	448.38	449.15	287.1	34	31	2.01

Isorhamnetin	$C_{16}H_{12}O_7$	316.28	315	151.0<br>300.2	43	30<br>20	2.86
Quercetin-3-O-rhamnoside	$C_{21}H_{20}O_{11}$	448.38	447.01	271.0<br>300.0	43	47<br>28	2.14
Quercetin-3-O-rutinoside	$C_{27}H_{30}O_{16}$	610.53	609.1	300.0<br>271	47	39<br>65	1.92
Hyperoside	$C_{21}H_{20}O_{12}$	464.38	463.3	300.0<br>271.15	47	24<br>44	1.99
Myricetin-3-galactoside	$C_{21}H_{20}O_{13}$	480.38	479.05	271.1<br>287.1	48	39<br>44	1.87
Kaempferol-3-O-rutinoside	$C_{21}H_{20}O_{10}$	432.39	431.05	255.3<br>284.2	45	42<br>28	2.27
Ipriflavone	$C_{18}H_{16}O_3$	280.33	281.3	240	40	19	4.17
Naringin	$C_{22}H_{32}O_{14}$	580.54	579.15	271.1<br>151.5	45	33<br>40	2.21

Table S3: Multiple Reaction Monitoring conditions for derivatized (into thioureas) isothiocyanates in UPLC-MS/MS analysis

(Isothiocyanate) derivatized thiourea	Chemical formula	Molecular weight	$[M-H]^+$ (m/z)	MS^2 fragments (m/z)	Cone voltage (V)	Collision energy (eV)	Retention time (R_t)
(Iberin) thiourea	$C_5H_{12}N_2OS_2$	180.29	181.3	163.4	22	15	4.61
(Allyl) thiourea	$C_4H_7N_2S$	115.18	116.18	99.8	19	13	6.13
(Sulforaphane) thiourea	$C_6H_{14}N_2OS_2$	194.31	195.4	117.2	12	11	3.22
(Benzyl) thiourea	$C_8H_{10}N_2S$	166.24	167.4	148.9	18	9	15.43
(Phenethyl) thiourea	$C_9H_{12}N_2S$	180.27	181.0	104.99	20	17	19.45
Indole-3-carbinol	C_9H_9NO	147.17	148.0	117	17	9	2.12

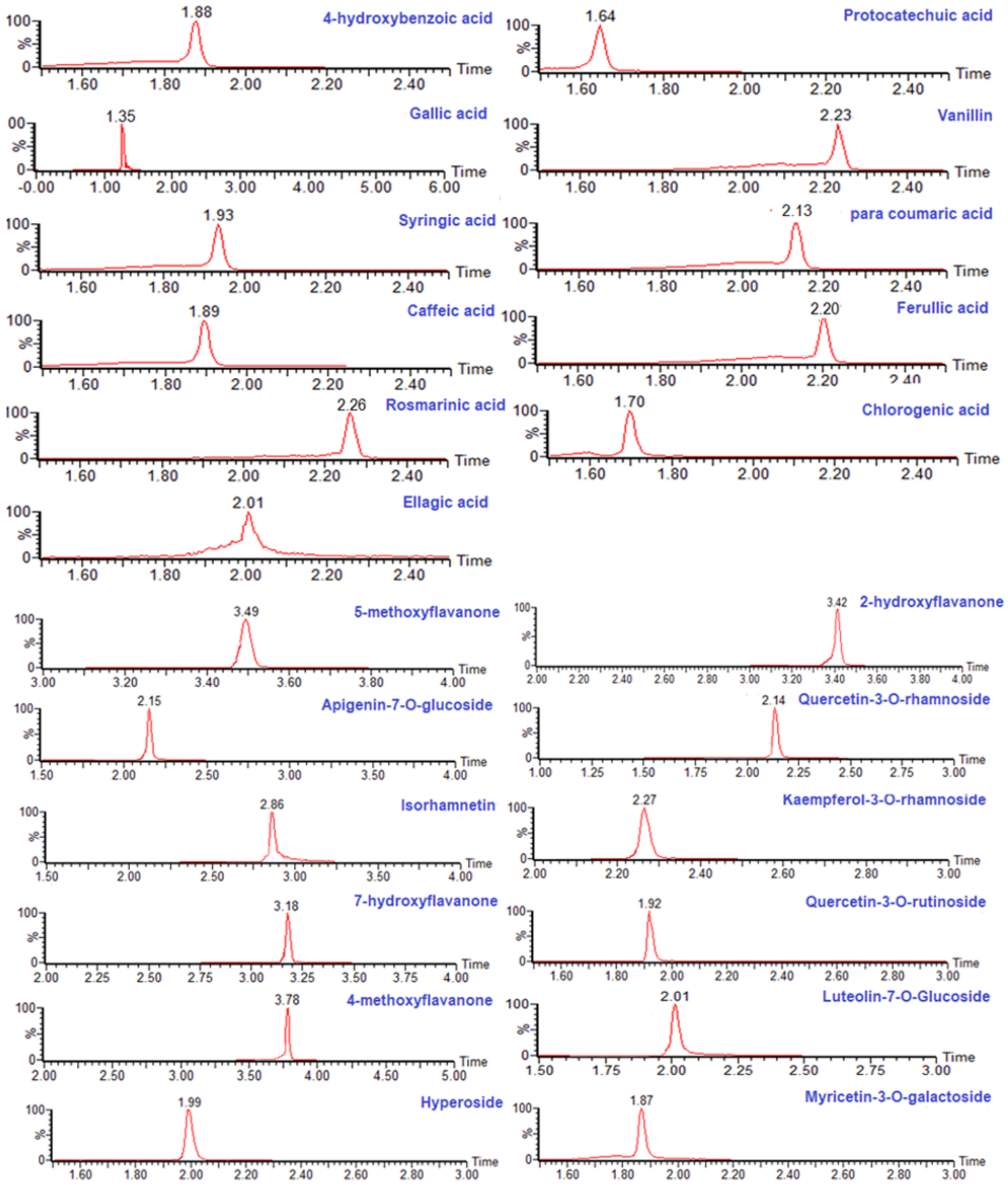

Figure S1: Extracted UPLC- ESI-MS/MS chromatograms of polyphenolic compounds (including phenolic acids and flavonoids) in watercress sample extracts.

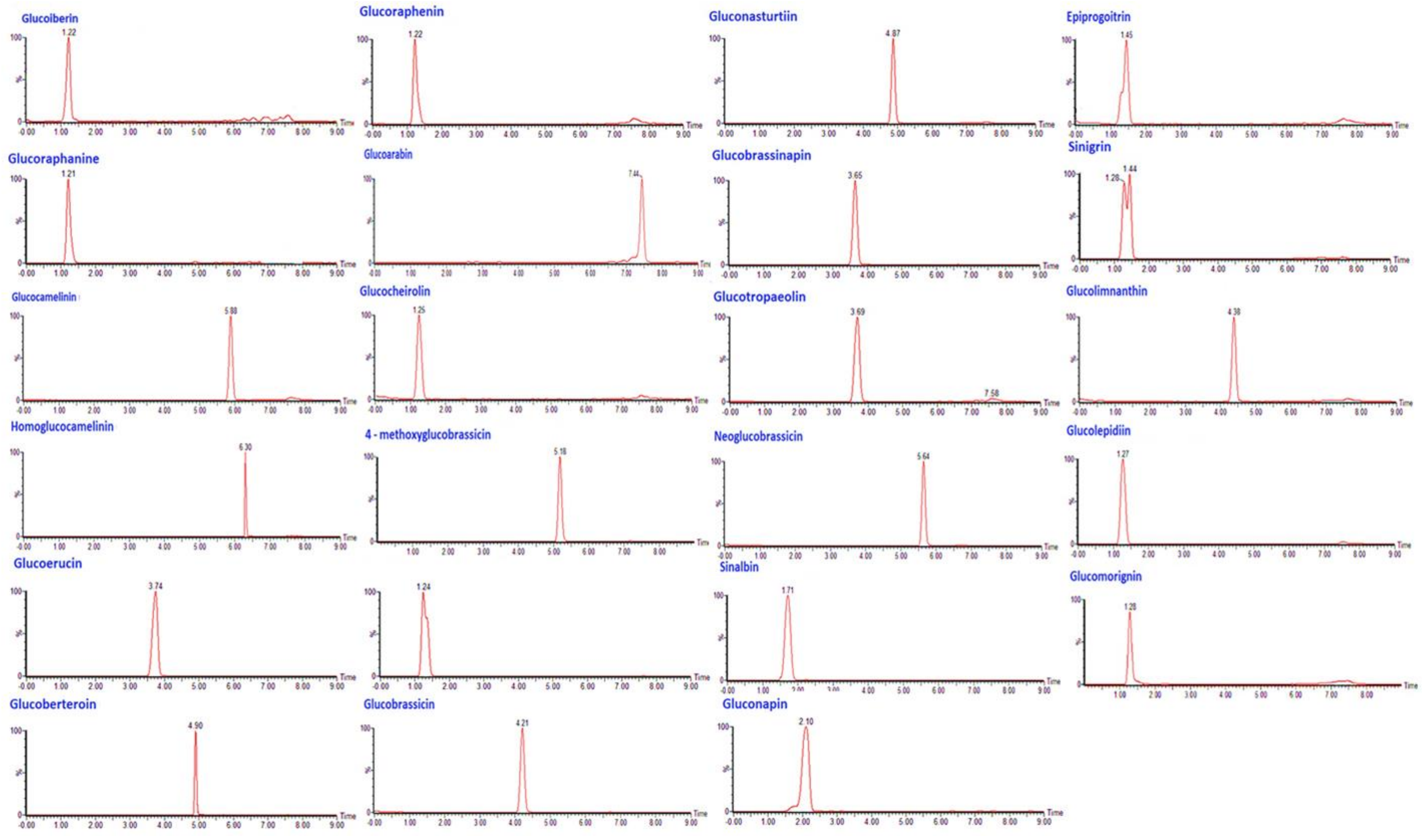

Figure S2: Extracted UPLC- ESI-MS/MS chromatograms of glucosinolates in watercress sample extracts.

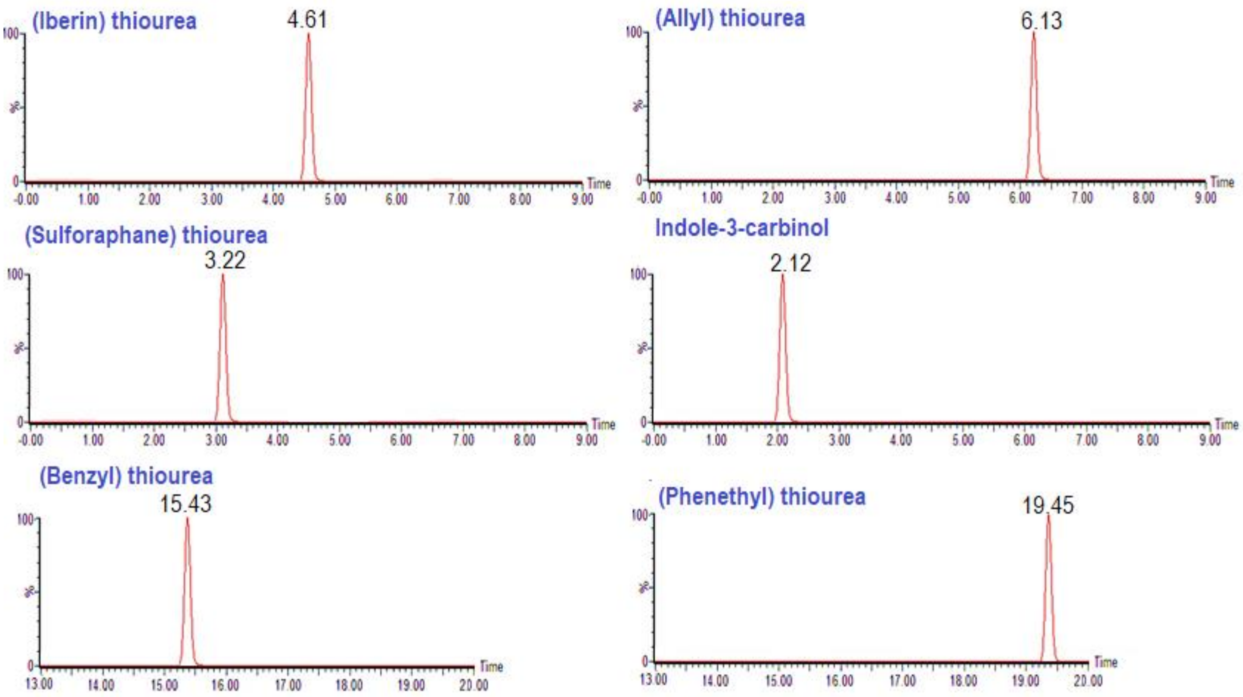

Figure S3: Extracted UPLC- ESI-MS/MS chromatograms of thioureas (derivatized isothiocyanates) in watercress sample extracts.

Table S4: The limit of detection (LOD), quantification (LOQ), linearity, precision and accuracy results for the screened intact glucosinolates. The calibration equations represent the peak area as a function of concentration in ppb. The intra- and inter- day experimental data concern data that have been collected from a six days experiment, whereas the %recovery data are the means of three indepented experiments.

Compound	Linear range (ppb)	LOD (ppb)	LOQ (ppb)	Calibration equation[a]	Correlation coefficient (r^2)	%RSD (intra-day)[b]	%RSD (inter-day)[c]	%REC[d]
				**GLUCOSINOLATES**				
Glucoiberin	0.27-249.80	0.27	0.79	y=0.21x-0.57	0.9998	0.98	2.28	98.3
Glucoraphanine	1.10-250.1	1.10	3.65	y=3.19x-4.6	0.9990	0.57	4.31	89.6
Glucocamelinin	1.69-250.10	1.69	5.64	y=4.54x-5.24	0.9991	0.65	2.65	84.0
Homoglucocamelinin	3.70-248.30	3.70	12.36	y=5.82x+1.04	0.9995	0.69	2.32	96.3
Glucoarabin	3.36-249.80	3.36	11.21	y=7.63x+2.22	0.9989	0.47	4.10	99.9
Glucoraphenine	5.22-246.60	5.22	17.40	y=2.41x-1.05	0.9989	0.58	1.95	100.2
Glucocheirolin	6.36-251.20	6.36	21.20	y=2.33x-5.00	0.9997	1.12	2.1	93.2
Glucolepidiin	4.26-248.60	4.26	14.20	y=1.13x-7.17	0.9954	1.32	2.53	85.0
Glucoberteroin	3.66-2.49	3.66	12.20	y=3.38x-6.27	0.9999	1.54	3.0	96.4
Glucoerucin	1.68-377.20	1.68	5.60	y=3.56x-2.64	0.9991	2.6	1.99	97.8
Sinigrin	1.96-255.40	1.96	6.54	y=1.46x-3.32	0.9981	2.2	1.1	98.4
Gluconapin	2.69-243.8	2.69	8.98	y=1.84x-1.48	0.9987	2.2	1.2	93.1
Glucobrassicanapin	4.37-246.5	4.37	14.58	y=1.16x-1.60	0.9992	2.3	1.03	87.6
Epiprogoitrin	6.79-237.40	6.79	22.65	y=0.98x-7.07	0.9995	1.89	1.04	99.5
Progoitrin	4.77-248.0	4.77	15.90	y=1.27x-0.58	0.9961	2.02	2.02	99.5
Glucolimnanthin	4.89-247.90	4.89	16.32	y=5.24x+0.93	0.9989	2.21	0.5	94.6
Sinalbin	5.36-262.30	5.36	17.89	y=2.25x-6.36	0.9932	1.87	1.37	89.9

[a]

Gluconasturtiin	2.99-258.20	2.99	9.98	y=2.08x-1.60	0.9977	2.25	4.2	95.5
Glucotropaeolin	2.69-250.50	2.69	8.97	y=7.07x+11.65	0.9983	1.14	0.78	92.2
Glucobrassicin	2.99-250.50	2.99	9.99	y=2.15x-2.44	0.9994	1.6	0.96	98.7
Glucomoringin	3.53-254.40	3.53	11.78	y=4.64x+3.73	0.9992	3.2	1.11	100.5
4-methoxyglucobrassicin	4.29-251.90	4.29	14.30	y=2.55x=1.21	0.9984	2.89	1.63	101.2
Neoglucobrassicin	3.75-233.90	3.75	12.50	y=8.014x-1.95	0.9993	1.21	2.42	93.7

Chromatographic peak area (y) as a function of ppb concentration (x)

[b] Values are means of intra-day assays (*n*=6)

[c] Values are means of inter-day assays (*n*=6)

[d] (*n*=3)

Table S5: The limit of detection (LOD), quantification (LOQ), linearity, precision and accuracy results for the screened polyphenolic compounds. The calibration equations represent the peak area as a function of concentration in ppb. The intra- and inter- day experimental data concern data that have been collected from a six days experiment, whereas the %recovery data are the means of three indepented experiments.

Compound	Linear range (ppb)	LOD (ppb)	LOQ (ppb)	Calibration equation[a]	Correlation coefficient (r^2)	%RSD		%REC[d]
						(intra-day)[b]	(inter-day)[c]	
**POLYPHENOLIC COMPOUNDS**								
4-hydroxybenzoic acid	3.01-499.50	3.01	14.20	y=36.87x-62.07	0.9991	1.15	2.21	98.8
Protocatechuic acid	0.66-504.50	0.66	14.70	y=34.24x-69.4	0.9995	1.25	2.65	86.3
Gallic acid	53.20-513.20	53.20	105.20	y=0.67x-1.5	0.9996	0.46	0.21	99.9
Vanillin	2.87-335.00	2.87	5.62	y=0.67x-0.1	0.9999	0.98	0.95	100.4
Syringic acid	2.01-501.60	2.01	2.86	y=7.28x-2.7	0.9996	1.36	1.01	96.6
p-coumaric acid	0.65-497.30	0.65	1.55	y=52.84x+36.9	0.9997	1.7	1.94	93.2
Caffeic acid	1.21-500	1.21	1.25	y=92.95x-344.4	0.9995	1.01	2.21	100.1
Ferulic acid	2.10-505.60	2.10	12. 17	y=19.02x-68.4	0.9992	0.7	2.45	102.6
Rosmarinic acid	2.32-499.50	2.32	2.56	y=7.03x+12.34	0.9996	1.3	3.02	86.9
Chlorogenic acid	3.48-495.60	3.48	4.76	y=25.02x+60.3	0.9991	1.35	1.98	87.4
Ellagic acid	5.53-499.10	5.53	75.60	y=2.18x+7.4	0.9995	1.32	3.05	89.9
2’-hydroxyflavanone	19.50-250.00	19.50	20.12	y=38.69x+22.5	0.9998	2.7	4.32	99.5

7-hydroxyflavanone	1.97-249.90	1.97	2.21	y=51.17x-73.6	1	2.63	1.42	98.9
4'-methoxyflavanone	2.21-250.00	2.21	3.89	y=83.54x+60.3	0.9999	2.89	1.87	93.6
5-methoxyflavanone	6.47-248.50	6.47	8.52	y=195.14x-493.9	0.9992	3.21	2.69	94.7
Apigenin-7-*O*-glucoside	1.87-125.30	1.87	4.42	y=6.17x+3.8	0.9998	3.48	2.54	95.8
Luteolin-7-*O*-glucoside	2.21-250.10	2.21	2.22	y=51.52x-89.9	0.9998	3.64	3.22	89.2
Isorhamnetin	14.01-251.1	14.01	2.31	y=6.08x-15.4	0.9992	2.48	1.18	100.1
Quercetin-3-*O*-rhamnoside	1.02-250.60	1.02	4.21	y=60.83x-38.6	0.9999	2.21	3.01	99.8
Quercetin-3-O-rutinoside	1.40-251.30	1.40	4.32	y=97.74x+109.7	0.9999	1.35	1.89	87.4
Hyperoside	6.32-249.90	6.32	3.21	y=3.97x+0.5	0.9998	2.14	1.37	96.3
Myricetin-3-galactoside	0.85-251.20	0.85	2.12	y=26.38x-31.8	0.9997	1.78	1.65	100.2
Kaempferol-3-*O*-rutinoside	0.76-250.00	0.76	1.21	y=25.73x+73.7	0.9997	1.36	2.21	91.2
Ipriflavone	109.90-250.00	109.90	13.21	y=0.62x+2.2	0.9994	1.69	1.11	93.6
Naringin	3.01-250.60	3.01	1.21	y=22.88x-43.3	0.9997	2.22	4.02	95.4

[a] Chromatographic peak area (y) as a function of ppb concentration (x)

[b] Values are means of intra-day assays (*n*=6)

[c] Values are means of inter-day assays (*n*=6)

[d] (*n*=3)

Table S6: The limit of detection (LOD), quantification (LOQ), linearity, precision and accuracy results for the screened derivatized isothiocyanates compounds. The calibration equations represent the peak area as a function of concentration in ppb. The intra- and inter- day experimental data concern data that have been collected from a six days experiment, whereas the %recovery data are the means of three indepented experiments.

**Compound**	**Linear range (ppb)**	**LOD (ppb)**	**LOQ (ppb)**	**Calibration equation**[a]	**Correlation coefficient (r^2)**	**%RSD**		**%REC**[d]
						**(intra-day)**[b]	**(inter-day)**[c]	
**(ISOTHIOCYANATE) DERIVATIZED THIOUREA COMPOUNDS**								
(Iberin) thiourea	21.69-250.50	21.69	72.32	y=3.12x+15	0.9999	1.18	4.02	84.2
(Allyl) thiourea	24.43-151.42	24.43	81.45	y=0.67x+1.1	0.9997	1.32	2.21	80.2
(Sulforaphane) thiourea	6.96-236.12	6.96	23.14	y=29.41x-36.9	0.9996	2.15	2.21	95.6
(Benzyl) thiourea	4.68-247.92	4.68	15.36	y=25.48x-65.1	0.9982	2.13	2.43	89.7
(Phenethyl) thiourea	3.06-250	3.06	10.21	y=48.12x-1.2	0.9997	2.22	2.6	96.7
Indole-3-carbinol	2.73-251.10	2.73	9.12	y=22.88x-43.3	0.9959	1.04	2.0	93.6

[a] Chromatographic peak area (y) as a function of ppb concentration (x)

[b] Values are means of intra-day assays (*n*=6)

[c] Values are means of inter-day assays (*n*=6)

[d] (*n*=3)

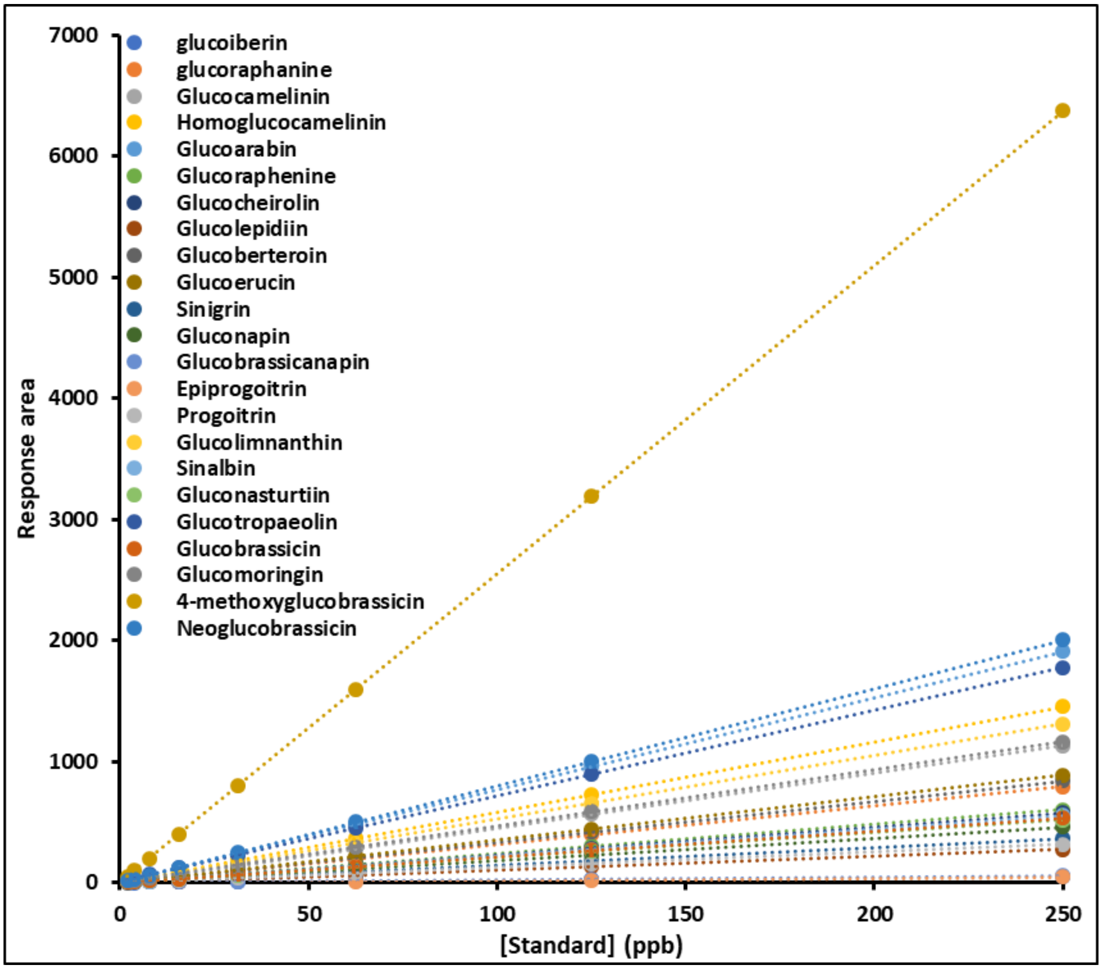

Figure S4: Calibration curve of glucosinolate standards at various concentrations (1.95-250 ppb) used for the determination of intact glucosinolates in the aerial parts (flowers, leaves and stems) of watercress.

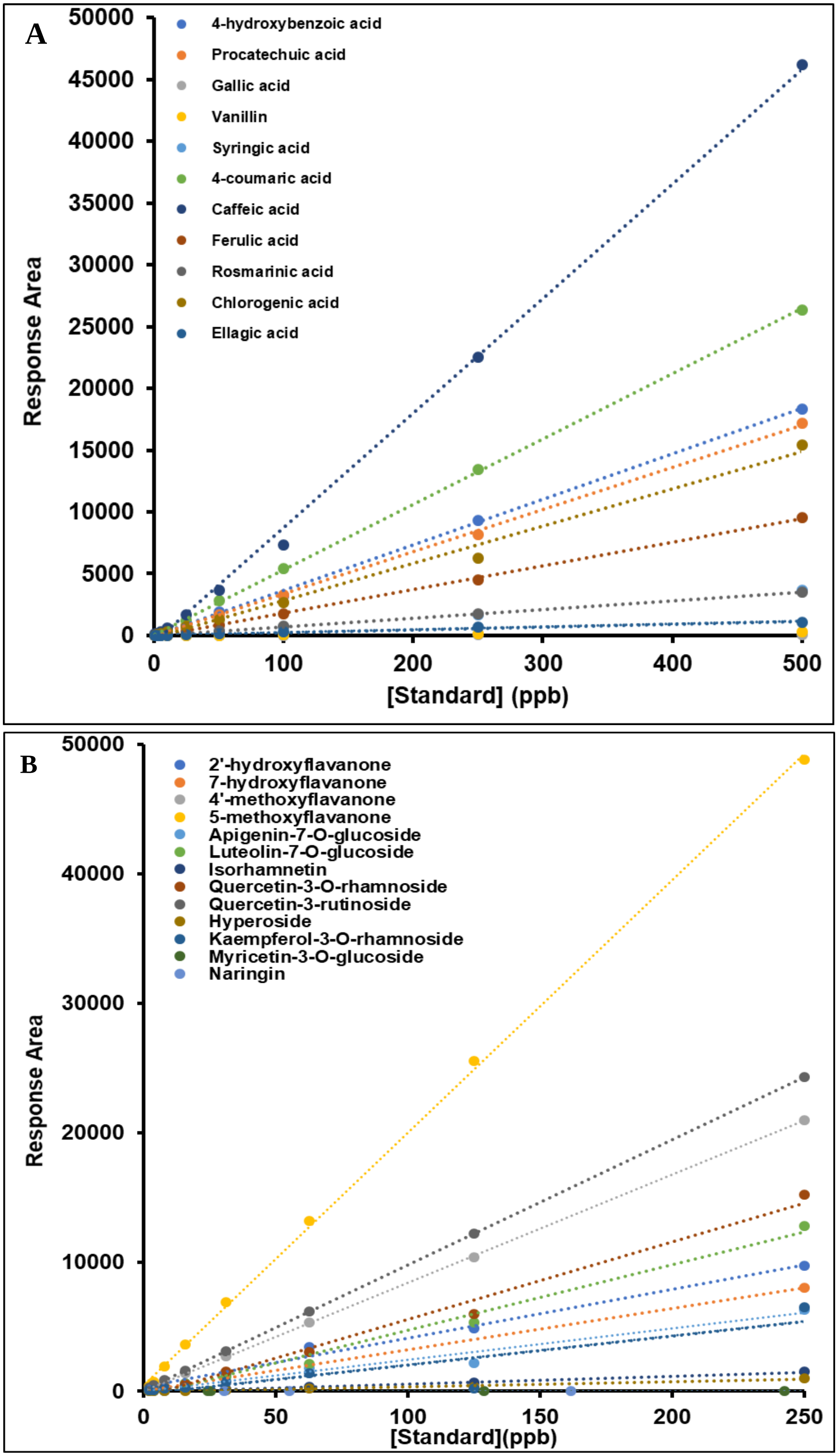
A
50000
45000
40000
35000
30000
25000
20000
15000
10000
5000
0
Response Area
0
100
200
300
400
500
[Standard] (ppb)
4-hydroxybenzoic acid
Procatechuic acid
Gallic acid
Vanillin
Syringic acid
4-coumaric acid
Caffeic acid
Ferulic acid
Rosmarinic acid
Chlorogenic acid
Ellagic acid
B
50000
40000
30000
20000
10000
0
Response Area
0
50
100
150
200
250
[Standard](ppb)
2'-hydroxyflavanone
7-hydroxyflavanone
4'-methoxyflavanone
5-methoxyflavanone
Apigenin-7-O-glucoside
Luteolin-7-O-glucoside
Isorhamnetin
Quercetin-3-O-rhamnoside
Quercetin-3-rutinoside
Hyperoside
Kaempferol-3-O-rhamnoside
Myricetin-3-O-glucoside
Naringin

Figure S5: Calibration curve of (**A**) phenolic acid and (**B**) flavonoid standards at various concentrations (1.95-500 ppb) used for the determination of polyphenolic compounds in the aerial parts (flowers, leaves and stems) of watercress.

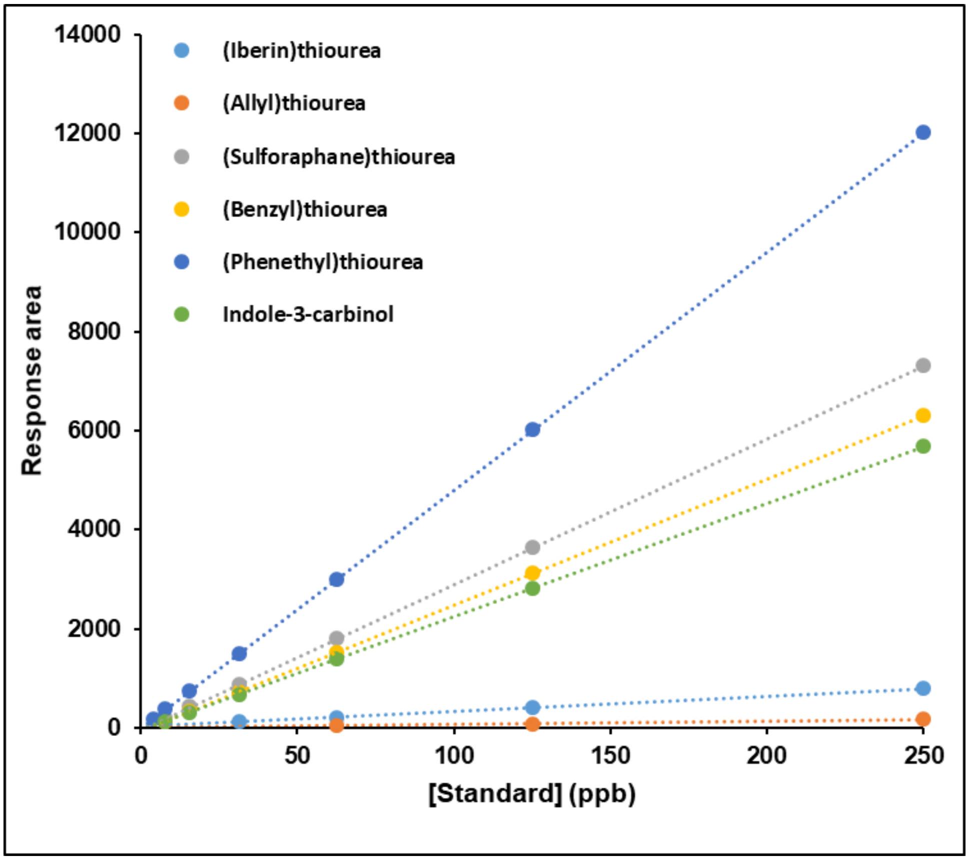

Figure S6: Calibration curve of derivatised isothiocyanates into the thiourea standards at various concentrations (1.95-250 ppb) used for the determination of isothiocyanates in the aerial parts (flowers, leaves.

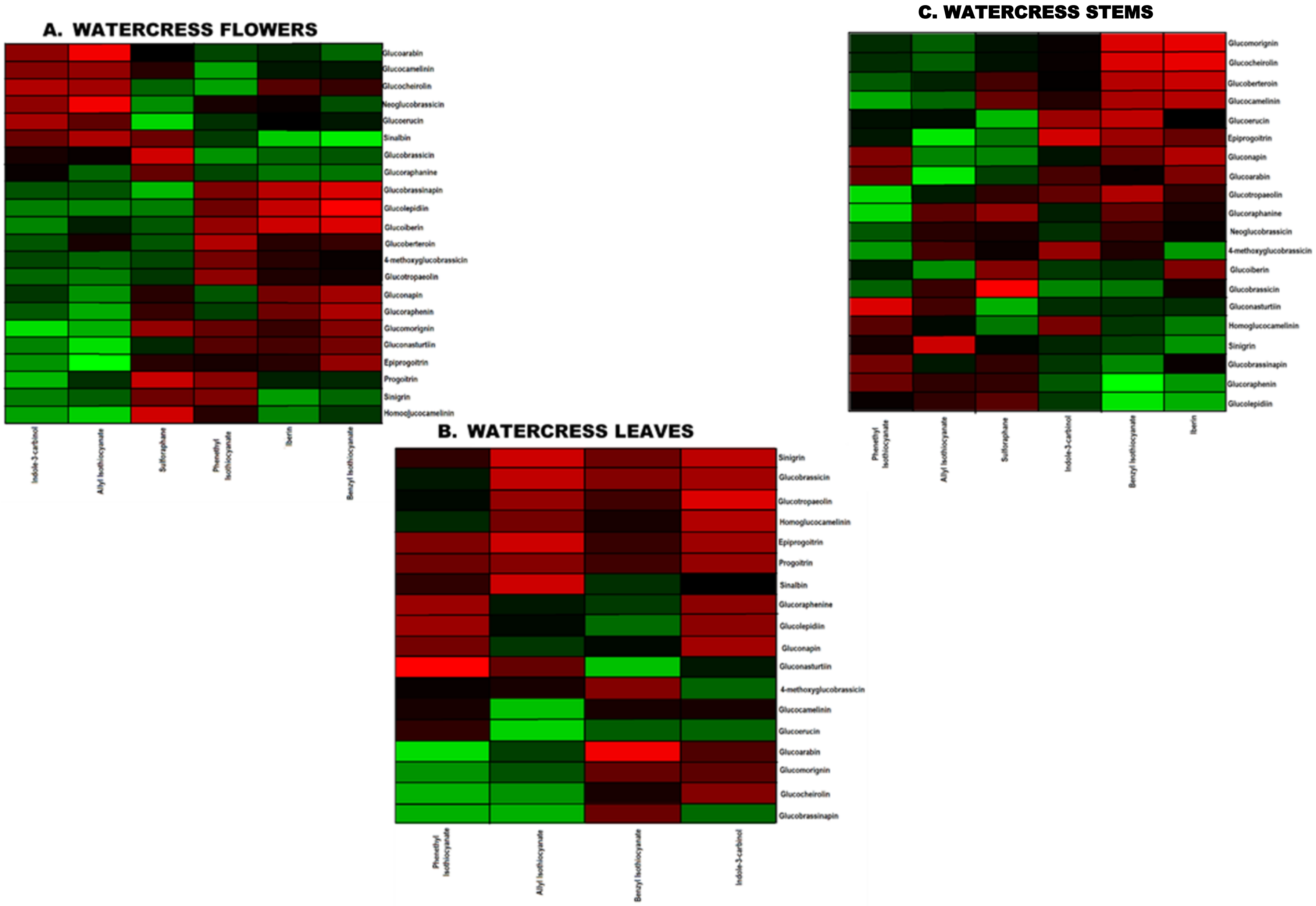
A. WATERCRESS FLOWERS
Glucoarabin
Glucocamelinin
Glucocheirolin
Neoglucobrassicin
Glucoerucin
Sinalbin
Glucobrassicin
Glucoraphanine
Glucobrassinapin
Glucolepidiin
Glucoiberin
Glucoberteroin
4-methoxyglucobrassicin
Glucotropaeolin
Gluconapin
Glucoraphenin
Glucomorignin
Gluconasturtiin
Epiprogoitrin
Progoitrin
Sinigrin
Homoglucocamelinin
Indole-3-carbinol
Allyl Isothiocyanate
Sulforaphane
Phenethyl Isothiocyanate
Iberin
Benzyl Isothiocyanate
B. WATERCRESS LEAVES
Sinigrin
Glucobrassicin
Glucotropaeolin
Homoglucocamelinin
Epiprogoitrin
Progoitrin
Sinalbin
Glucoraphenine
Glucolepidiin
Gluconapin
Gluconasturtiin
4-methoxyglucobrassicin
Glucocamelinin
Glucoerucin
Glucoarabin
Glucomorignin
Glucocheirolin
Glucobrassinapin
Phenethyl Isothiocyanate
Allyl Isothiocyanate
Benzyl Isothiocyanate
Indole-3-carbinol
C. WATERCRESS STEMS
Glucomorignin
Glucocheirolin
Glucoberteroin
Glucocamelinin
Glucoerucin
Epiprogoitrin
Gluconapin
Glucoarabin
Glucotropaeolin
Glucoraphanine
Neoglucobrassicin
4-methoxyglucobrassicin
Glucoiberin
Glucobrassicin
Gluconasturtiin
Homoglucocamelinin
Sinigrin
Glucobrassinapin
Glucoraphenin
Glucolepidiin
Phenethyl Isothiocyanate
Allyl Isothiocyanate
Sulforaphane
Indole-3-carbinol
Benzyl Isothiocyanate
Iberin

Figure S7: Spearman's coefficient analysis between intact GL and ITC contents on watercress (A) flowers, (B) stems and (C) leaves. Red indicates positive (+1.0) correlation while green indicate a negative correlation. Black suggests no correlation.
